# An Anatomical Description of a Miniaturized Acorn Worm (Hemichordata, Enteropneusta) with Asexual Reproduction by Paratomy

**DOI:** 10.1371/journal.pone.0048529

**Published:** 2012-11-07

**Authors:** Katrine Worsaae, Wolfgang Sterrer, Sabrina Kaul-Strehlow, Anders Hay-Schmidt, Gonzalo Giribet

**Affiliations:** 1 Marine Biological Section, Department of Biology, University of Copenhagen, Copenhagen, Denmark; 2 Bermuda Natural History Museum, Flatts, Bermuda; 3 Department for Molecular Evolution and Development, University of Vienna, Vienna, Austria; 4 Department of Neuroscience and Pharmacology, The Panum Institute, University of Copenhagen, Copenhagen, Denmark; 5 Museum of Comparative Zoology, Department of Organismic and Evolutionary Biology, Harvard University, Cambridge, Massachussetts, United States of America; Sars International Centre for Marine Molecular Biology, Norway

## Abstract

The interstitial environment of marine sandy bottoms is a nutrient-rich, sheltered habitat whilst at the same time also often a turbulent, space-limited, and ecologically challenging environment dominated by meiofauna. The interstitial fauna is one of the most diverse on earth and accommodates miniaturized representatives from many macrofaunal groups as well as several exclusively meiofaunal phyla. The colonization process of this environment, with the restrictions imposed by limited space and low Reynolds numbers, has selected for great morphological and behavioral changes as well as new life history strategies.

Here we describe a new enteropneust species inhabiting the interstices among sand grains in shallow tropical waters of the West Atlantic. With a maximum body length of 0.6 mm, it is the first microscopic adult enteropneust known, a group otherwise ranging from 2 cm to 250 cm in adult size. Asexual reproduction by paratomy has been observed in this new species, a reproductive mode not previously reported among enteropneusts.

Morphologically, *Meioglossus psammophilus* gen. et sp. nov. shows closest resemblance to an early juvenile stage of the acorn worm family Harrimaniidae, a result congruent with its phylogenetic placement based on molecular data. Its position, clearly nested within the larger macrofaunal hemichordates, suggests that this represents an extreme case of miniaturization. The evolutionary pathway to this simple or juvenile appearance, as chiefly demonstrated by its small size, dense ciliation, and single pair of gill pores, may be explained by progenesis. The finding of *M. psammophilus* gen. et sp. nov. underscores the notion that meiofauna may constitute a rich source of undiscovered metazoan diversity, possibly disguised as juveniles of other species.

## Introduction

Hemichordata is a phylum of exclusively marine deuterostomes traditionally divided into the classes Pterobranchia and Enteropneusta [1]–[4], the latter including Ptychoderidae, Spengeliidae, Harrimaniidae and Torquaratoridae e.g., [4], [5]. However, Enteropneusta is in several recent studies [6], [7], though not all [8], considered paraphyletic with respect to the Pterobranchia. Whereas all known acorn worms are solitary and range from 2–250 cm in adult size, constituting part of the benthic macro-infauna, the sessile pterobranchs are colonial and consist of multiple minute zooids, each usually 0.5–5 mm long [9]–[11]. The origin of this deviant and small body plan is still poorly understood, especially if they nest among clades of macroscopic solitary acorn worms [6], [7]. It is thus inferred that a pterobranch ancestor shared traits with these and may have shown resemblance to a solitary, mobile acorn worm [6], [7].

The meiofauna comprises free-living animals that can pass a 1 mm (0.5 mm) sieve but are retained on a 31 µm (44 µm) sieve [12], [13]. Metazoan meiofauna is dominated by protostomes, including miniaturized representatives of Annelida, Arthropoda, and Mollusca, as well as a variety of mostly meiofaunal lineages such as Gastrotricha, Kinorhyncha, many Platyhelminthes groups, Rotifera and Tardigrada [12]. Deuterostomia, the other major bilaterian clade, consists of three major extant lineages—Echinodermata, Hemichordata, Chordata and arguably Acoelomorpha (including Xenoturbellida) [14]–[21]. Most deuterostome species are macrofaunal, while microscopic, meiofaunal representatives of the deuterostome lineages are restricted to a handful of Tunicata (Chordata), Holothuroidea (Echinodermata), and, if truly deuterostome, most acoelomorphs [22]–[30].

Most remarkable is the phylogenetic diversity and disparity in body plans found in this size category, wherefrom three of the most recent phyla Gnathostomulida, Loricifera, and Micrognathozoa were described [31]–[35].This fraction of small-sized metazoans is extraordinarily numerous and diverse in the interstices among sand grains, an environment that includes representatives of more than twenty of the thirty-some animal phyla [12]–[13]. This hidden and often overlooked source of diversity was highlighted in recent meiofaunal surveys from two of the best-studied shallow water localities in the North Sea and the Mediterranean, with up to 37% of the reported species being undescribed [36], [37]. The use of genetic tools has also aided to identify cryptic species in this environment [38]–[41], amplifying the magnitude of unknown meiofaunal biodiversity. However, to date no adult acorn worm had been reported from the interstitial environment.

Meiofaunal animals may have been major players in the earliest stages of the diversification of bilaterian life [13], [42]. Many others became miniaturized secondarily from macrofaunal ancestors [43]–[46]. The most popular evolutionary hypotheses explaining the many miniaturized forms in the interstitial environment, including meiofaunal ascidians and holothurians, is progenesis [26], [29], [47], based mainly on their resemblance to larval or juvenile stages of their macrofaunal relatives. A second argument in favor of progenesis has to do with the difficulties in adapting to the interstitial environment, requiring one macroevolutionary change rather than a gradual decrease in size [43], [47]. With an inherited early offset of somatic growth in a young life stage facilitated by, e.g., early or fast maturation, a one-step evolutionary pathway is accessed (progenesis); leading to the permanent colonization of the interstitial habitat. Paedomorphosis (mainly progenesis rather than neoteny) is therefore suggested to be the most likely evolutionary pathway for the origin of many interstitial taxa, especially those with extant macrofaunal relatives [43], [45], [47]–[52]. This is possible since many macrofaunal species, including enteropneusts [53], [54], already spend part of their life cycle in the interstitial environment as newly settled larvae and juveniles growing up in this protected habitat [12], [13].

Hemichordata present a variety of reproductive modes including different forms of asexual reproduction [55]–[57], which, though possibly homologous, may add to the complexity of tracing their evolutionary history and address theories such as progenesis. The most common form of asexual reproduction in Enteropneusta is architomy (fission/fragmentation followed by regeneration of individuals). This is described as a transverse fission leading to a posterior (hepatic) regenerating individual and an anterior (branchio-genital) individual [55]. The posterior genital part of the anterior branchio-genital individual fragments into several ‘regenerates’ (zooids) that regenerate into new individuals post fission [58], [59]. Paratomy is an alternative to architomy and common in, e.g., Annelida and Acoela [60]–[62]. It is described as transverse fission following a complete regeneration of organ systems in individuals developing in alignment with the anterior-posterior body axis, head to tail fashion [62]. Budding is common in the colonial Pterobranchia (Hemichordata) and can in, e.g., *Cephalodiscus*, be regarded as a special form of ‘paratomy’ where the axis of the new zooid does not correspond to the axis of the old zooid, yet may detach [55], [63]. In *Rhabdopleura* the bud may develop along the anteroposterior axis to full maturity [55], [57], however a bud has never been observed detaching from the mother zooid.

Here we report the first meiofaunal, interstitial adult acorn worm and provide a detailed description of its anatomy and possible reproduction mode. We further test its phylogenetic position using DNA sequence data and standard phylogenetic analyses. Possible evolutionary scenarios for this, the smallest acorn worm known to date, are discussed.

## Results

### Taxonomic treatment

Phylum Hemichordata Bateson, 1885

Class Enteropneusta Gegenbaur, 1870

Family Harrimaniidae Spengel, 1901

Genus *Meioglossus* gen. nov.

#### Diagnosis

Interstitial, microscopic enteropneust with slender, completely ciliated body. Proboscis elongated; with a pair of dorso-lateral proboscis pores at the base; indistinct neck. Longitudinal blood vessel running underneath the collar cord and projecting into the protocoel, to enlarge into glomerulus. Pericardium small; attached postero-dorsally to the wall of the protocoel and ventrally to the glomerulus; indistinct heart sinus. Stomochord and nuchal skeleton absent. Collar region more than half the length of proboscis; with paired mesocoels separated by longitudinal septa; mesocoelic pores absent. Perihaemal cavities present, terminating at base of proboscis. Peribuccal cavities absent. Trunk region with one pair of anterior dorso-lateral gill pores. Mesocoels and metacoels separated by a transverse septum, anterior to the gill pores, at the level of the posterior collar ridge.

#### Etymology

From Greek, *meio*-, smaller and *glossa*, tongue, indicating the size of this species (-*glossus* is typically used in enteropneust genera, such as *Glossobalanus*, *Balanoglossus*).

*Meioglossus psammophilus* sp. nov.

**Figure 1:**
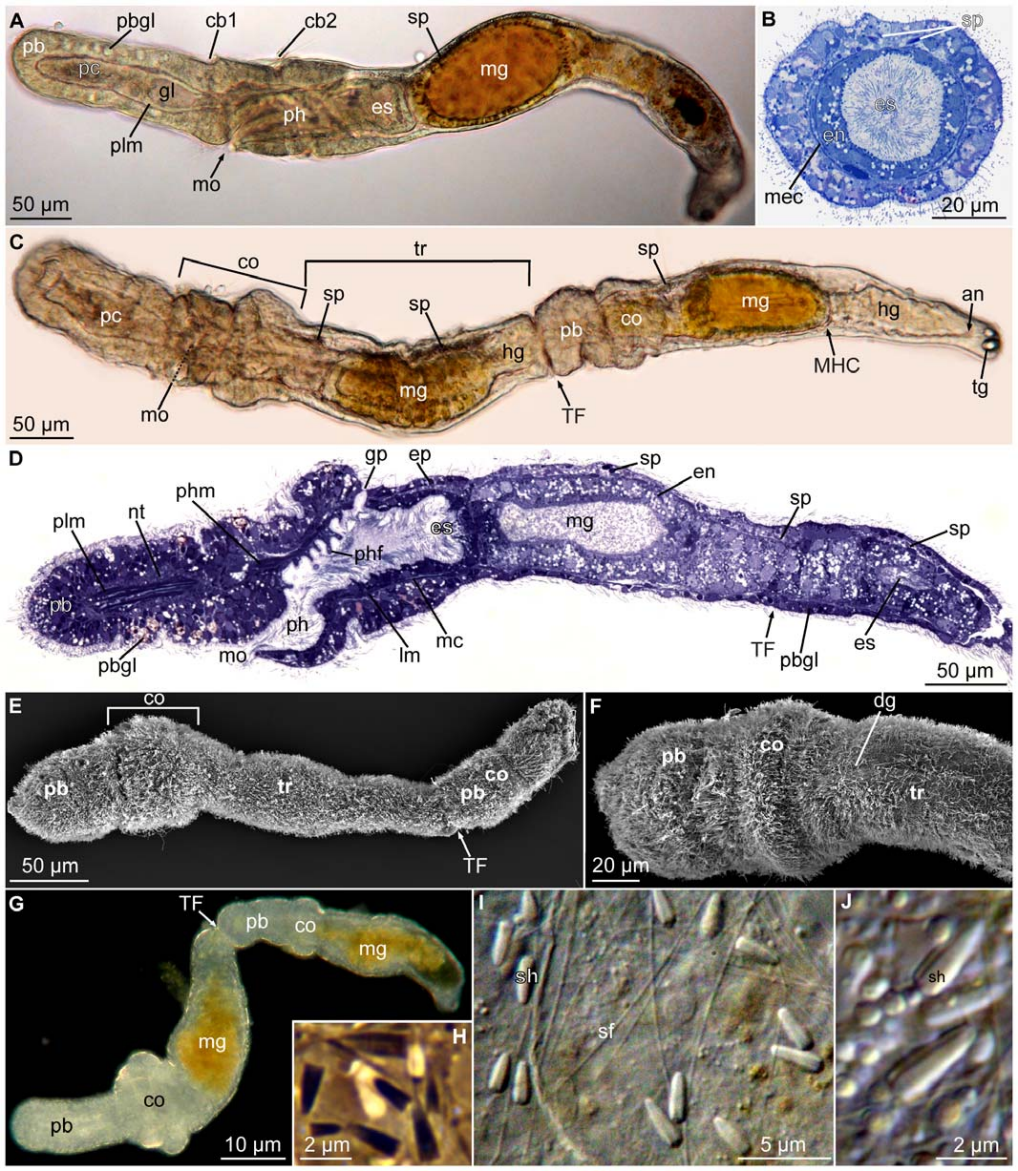
*Meioglossus psammophilus* gen. et sp. nov., light- and scanning electron micrographs (LM and SEM). A. Overview of specimen, lateral view, LM (bright field, DIC). B. Cross section of anterior trunk region showing dorsal position of sperm, LM (bright field). C. Dorsal live view, paratomy (transverse fission, two individuals) and sperm, LM (bright field). D. Semithin sagittal section, toluidine blue stained, LM. E. Specimen showing paratomy, lateral view, SEM (ZMUC-ENP-6). F. Close up from E of anterior end, dorsal view, SEM. G. Specimen showing paratomy, dorsal live view, LM (dark field). H. Different shapes and sizes of sperm heads from paratomy specimen in G, LM (phase contrast). I. Sperm heads and flagella, LM (bright field, DIC). J. Close up of sperm heads from I. Abbreviations: an, anus; cb1, cb2, 1. & 2. ciliary band; co, collar region; en, endodermal cell; ep, epidermis; es, esophagus; gl, glomerulus; gp, gill pore; hg, hindgut; lm, longitudinal muscles; mc, mesocoel; mec, metacoel; mg, midgut; MHC, midgut-hindgut constriction; mo, mouth opening; nt, nerve tissue; pb, proboscis; pbgl, proboscis gland; pc, protocoel; ph, pharynx; phf, pharyngeal fold; phm, perihaemal muscles; plm, proboscis longitudinal muscles; sf, sperm flagellum; sh, sperm head; sp, sperm; TF, transverse fission zone; tr, trunk; tg, tail glands.

*Glossobalanus crozieri* van der Horst, 1924 (juvenile): Tyler 2001 [64]: 3–12 (Figure 1).

#### Diagnosis

Body hyaline with yellow gut. Proboscis delineated posteriorly by a circumferential ring of cilia. Protocoel extending most of the length and width of the proboscis; lined by a one-fiber thick ring of longitudinal muscles, encircled by circular musculature. Collar with middle circumferential groove lined by a ring of long cilia and epidermal glands. Dorsal collar cord running subepidermally, neuropores absent. Anterior trunk with slight longitudinal depression mid-dorsally; one pair of anterior dorso-lateral gill pores lacking the characteristic ciliation of the distal part of the gill pores. Posterior trunk with supraterminal anus and large terminal glands. Sexual reproduction likely; with sperm in paired testes or free in metacoel. Asexual reproduction by transverse fission, in the form of paratomy.

#### Etymology

From Greek, *psammon-*, sand, and *philos*, friend, referring to the interstitial life habit of the adults of this species.

#### Holotype

Male, 611 µm long, from coral sand at reef, 15 m depth, Carrie Bow Cay, Belize (16°48.127′N, 88°04.607′W), leg. K. Worsaae, 23 January 2010, deposited in Natural History Museum, Denmark (SNM) (wholemount, ZMUC-ENP-1).

#### Paratypes

Forty-one specimens (males only) from coral sand at reef, 15 m depth, Carrie Bow Cay, Belize (16°48.2′N, 88°04.6′W), April 2009 and January 2010, leg. K. Worsaae (ZMUC-ENP-2 to ZMUC-ENP-42). Forty-one specimens (no sexually mature) from coral sand at reef, 2–12 m depth, Windsor Beach and Harrington sound, Bermuda (32°19.9′N 64°40.6′W; 32°19.95′N, 64°43.69′W), October 2007, leg. K. Worsaae & W. Sterrer (ZMUC-ENP-43 to ZMUC-ENP-83); none of these with sperm whereas a previous June 2005 collecting in Bermuda yielded several males (not preserved). All material deposited at the Natural History Museum, Denmark (SNM) (30 specimens as permanent whole mounts, 16 on SEM stubs, 8 sectioned on slides or grids, 9 embedded in resin, as well as 10 vials with aldehyde-fixed specimens, 9 vials with etoh-fixed specimens; see Table 1 for details). Additional animals were photographed and recorded alive, and due to squeezing discarded afterwards or fixed for DNA-extraction. Roughly one third of the examined species exhibited paratomy. DNA sequence data were obtained from one entire specimen from Windsor Beach, Bermuda.

**Table 1:** Type material and no. of specimens examined with different methods in the presented study.

Catalogue No.	Storage medium	Fixation	Preparation	Staining	Sex	Water depth (m)	Date	Coordinates	Legit
ZMUC-ENP-1	WM (holotype)	PFA	CLSM	tyr-tub, ser, dapi	male	15	23.01.2010	16°48.127′N, 88°04.607′W	KW
ZMUC-ENP-2	WM	PFA	CLSM	α-tub, FMRF, dapi	male	15	23.01.2010	16°48.127′N, 88°04.607′W	KW
ZMUC-ENP-3	WM	PFA	CLSM	α-tub, ser, phal, dapi	male	15	23.01.2010	16°48.127′N, 88°04.607′W	KW
ZMUC-ENP-4	WM	PFA	CLSM	α-tub, FMRF, dapi	male	15	23.01.2010	16°48.127′N, 88°04.607′W	KW
ZMUC-ENP-5	WM	PFA	CLSM	α-tub, FMRF, dapi	male	15	23.01.2010	16°48.127′N, 88°04.607′W	KW
ZMUC-ENP-6	WM	PFA	CLSM	tyr-tub, ser, dapi	male	15	23.01.2010	16°48.127′N, 88°04.607′W	KW
ZMUC-ENP-7	stub	1% OsO4	SEM			15	23.01.2010	16°48.127′N, 88°04.607′W	KW
ZMUC-ENP-8	stub	1% OsO4	SEM			15	23.01.2010	16°48.127′N, 88°04.607′W	KW
ZMUC-ENP-9	stub	1% OsO4	SEM			15	23.01.2010	16°48.127′N, 88°04.607′W	KW
ZMUC-ENP-10	stub	1% OsO4	SEM			15	23.01.2010	16°48.127′N, 88°04.607′W	KW
ZMUC-ENP-11	stub	1% OsO4	SEM			15	23.01.2010	16°48.127′N, 88°04.607′W	KW
ZMUC-ENP-12	stub	1% OsO4	SEM			15	23.01.2010	16°48.127′N, 88°04.607′W	KW
ZMUC-ENP-13	stub	1% OsO4	SEM			15	23.01.2010	16°48.127′N, 88°04.607′W	KW
ZMUC-ENP-14	stub	1% OsO4	SEM			15	23.01.2010	16°48.127′N, 88°04.607′W	KW
ZMUC-ENP-15	stub	1% OsO4	SEM			15	23.01.2010	16°48.127′N, 88°04.607′W	KW
ZMUC-ENP-16	stub	1% OsO4	SEM			15	23.01.2010	16°48.127′N, 88°04.607′W	KW
ZMUC-ENP-17	stub	1% OsO4	SEM			15	23.01.2010	16°48.127′N, 88°04.607′W	KW
ZMUC-ENP-18	resin	GLU	none			15	20.01.2010	16°48.127′N, 88°04.607′W	KW
ZMUC-ENP-19	resin	GLU	none			15	20.01.2010	16°48.127′N, 88°04.607′W	KW
ZMUC-ENP-20	resin	GLU	none			15	20.01.2010	16°48.127′N, 88°04.607′W	KW
ZMUC-ENP-21	resin	GLU	none			15	20.01.2010	16°48.127′N, 88°04.607′W	KW
ZMUC-ENP-22	resin	GLU	none			15	20.01.2010	16°48.127′N, 88°04.607′W	KW
ZMUC-ENP-23	resin	GLU	none			15	23.01.2010	16°48.127′N, 88°04.607′W	KW
ZMUC-ENP-24	resin	GLU	none			15	23.01.2010	16°48.127′N, 88°04.607′W	KW
ZMUC-ENP-25	resin	GLU	none			15	23.01.2010	16°48.127′N, 88°04.607′W	KW
ZMUC-ENP-26	resin	GLU	none			15	23.01.2010	16°48.127′N, 88°04.607′W	KW
ZMUC-ENP-27	resin (slides)	GLU	Hist	TB	male	15	23.01.2010	16°48.127′N, 88°04.607′W	KW
ZMUC-ENP-28	resin (slides)	GLU	Hist	TB	male	15	23.01.2010	16°48.127′N, 88°04.607′W	KW
ZMUC-ENP-29	resin (grids and slides)	GLU	Hist & TEM	TB or UA+LC	male	15	23.01.2010	16°48.127′N, 88°04.607′W	KW
ZMUC-ENP-30	resin (slides)	GLU	Hist	TB	male	15	09.04.2009	16°48′N, 88°04′W	WS
ZMUC-ENP-31	resin (grids and slides)	GLU	Hist & TEM	TB or UA+LC	male	15	23.01.2010	16°48.127′N, 88°04.607′W	KW
ZMUC-ENP-32	resin (grids and slides)	GLU	Hist & TEM	TB or UA+LC	male	15	23.01.2010	16°48.127′N, 88°04.607′W	KW
ZMUC-ENP-33	resin (grids and slides)	GLU	Hist & TEM	TB or UA+LC	male	15	23.01.2010	16°48.127′N, 88°04.607′W	KW
ZMUC-ENP-34	resin (grids and slides)	GLU	Hist & TEM	TB or UA+LC	male	15	23.01.2010	16°48.127′N, 88°04.607′W	KW
2 further specimens examined	grids lost	GLU	TEM	UA+LC	male	15	23.01.2010	16°48.127′N, 88°04.607′W	KW
ZMUC-ENP-35	GLU (ca. 5 specimens)	GLU	none			15	16.01.2010	16°48.224′N, 88°04.615′W	KW
ZMUC-ENP-36	70% etoh (ca. 1 specimen)	GLU	none			15	23.01.2010	16°48.127′N, 88°04.607′W	KW
ZMUC-ENP-37	GLU (ca. 7 specimens)	GLU	none			15	23.01.2010	16°48.127′N, 88°04.607′W	KW
ZMUC-ENP-38	PBS+NaN_3_ (ca. 20 specimens)	PFA	none			15	23.01.2010	16°48.127′N, 88°04.607′W	KW
ZMUC-ENP-39	96% etoh (4 specimens)	96% etoh	none			15	16.01.2010	16°48.224′N, 88°04.615′W	KW
ZMUC-ENP-40	96% etoh (6 specimens)	96% etoh	none			15	16.01.2010	16°48.224′N, 88°04.615′W	KW
ZMUC-ENP-41	96% etoh (5 specimens)	96% etoh	none			15	16.01.2010	16°48.224′N, 88°04.615′W	KW
ZMUC-ENP-42	96% etoh (6 specimens)	96% etoh	none			15	20.01.2010	16°48.127′N, 88°04.607′W	KW
ZMUC-ENP-43	WM	PFA	CLSM	α-tub, ser, phal, dapi		12	18.10.2007	32°19.9′N 64°40.6′W	KW & WS
ZMUC-ENP-44	WM	PFA	CLSM	α-tub, FMRF, dapi		12	18.10.2007	32°19.9′N 64°40.6′W	KW & WS
ZMUC-ENP-45	WM	PFA	CLSM	phal, dapi		12	18.10.2007	32°19.9′N 64°40.6′W	KW & WS
ZMUC-ENP-46	WM	PFA	CLSM	α-tub, FMRF, dapi		12	18.10.2007	32°19.9′N 64°40.6′W	KW & WS
ZMUC-ENP-47	WM	PFA	CLSM	phal, dapi		12	18.10.2007	32°19.9′N 64°40.6′W	KW & WS
ZMUC-ENP-48	WM	PFA	CLSM	α-tub, FMRF, dapi		12	18.10.2007	32°19.9′N 64°40.6′W	KW & WS
ZMUC-ENP-49	WM	PFA	CLSM	α-tub, FMRF, dapi		12	18.10.2007	32°19.9′N 64°40.6′W	KW & WS
ZMUC-ENP-50	WM	PFA	CLSM	α-tub, ser, phal, dapi		12	18.10.2007	32°19.9′N 64°40.6′W	KW & WS
ZMUC-ENP-51	WM	PFA	CLSM	α-tub, ser, phal, dapi		12	18.10.2007	32°19.9′N 64°40.6′W	KW & WS
ZMUC-ENP-52	WM	PFA	CLSM	α-tub, ser, phal, dapi		12	18.10.2007	32°19.9′N 64°40.6′W	KW & WS
ZMUC-ENP-53	WM	PFA	CLSM	tyr-tub, ser, phal, dapi		12	18.10.2007	32°19.9′N 64°40.6′W	KW & WS
ZMUC-ENP-54	WM	PFA	CLSM	tyr-tub, ser, phal, dapi		12	18.10.2007	32°19.9′N 64°40.6′W	KW & WS
ZMUC-ENP-55	WM	PFA	CLSM	tyr-tub, ser, phal, dapi		12	18.10.2007	32°19.9′N 64°40.6′W	KW & WS
ZMUC-ENP-56	WM	PFA	CLSM	tyr-tub, ser, phal, dapi		12	18.10.2007	32°19.9′N 64°40.6′W	KW & WS
ZMUC-ENP-57	WM	PFA	CLSM	tyr-tub, ser, phal, dapi		12	18.10.2007	32°19.9′N 64°40.6′W	KW & WS
ZMUC-ENP-58	WM	PFA	CLSM	tyr-tub, ser, phal, dapi		12	18.10.2007	32°19.9′N 64°40.6′W	KW & WS
ZMUC-ENP-59	WM	PFA	CLSM	tyr-tub, ser, phal, dapi		12	18.10.2007	32°19.9′N 64°40.6′W	KW & WS
ZMUC-ENP-60	WM	PFA	CLSM	α-tub, ser, phal, dapi		12	18.10.2007	32°19.9′N 64°40.6′W	KW & WS
ZMUC-ENP-61	WM	PFA	CLSM	α-tub, ser, dapi		12	18.10.2007	32°19.9′N 64°40.6′W	KW & WS
ZMUC-ENP-62	WM	PFA	CLSM	α-tub, ser, phal, dapi		12	18.10.2007	32°19.9′N 64°40.6′W	KW & WS
ZMUC-ENP-63	WM	PFA	CLSM	α-tub, ser, phal, dapi		12	18.10.2007	32°19.9′N 64°40.6′W	KW & WS
ZMUC-ENP-64	WM	PFA	CLSM	α-tub, ser, dapi		12	18.10.2007	32°19.9′N 64°40.6′W	KW & WS
ZMUC-ENP-65	WM	PFA	CLSM	α-tub, FMRF, dapi		12	18.10.2007	32°19.9′N 64°40.6′W	KW & WS
ZMUC-ENP-66	WM	PFA	CLSM	tyr-tub, phal, dapi		12	18.10.2007	32°19.9′N 64°40.6′W	KW & WS
ZMUC-ENP-67	WM	PFA	CLSM	α-tub, FMRF, dapi		12	18.10.2007	32°19.9′N 64°40.6′W	KW & WS
ZMUC-ENP-68	stub	1% OsO4	SEM			12	18.10.2007	32°19.9′N 64°40.6′W	KW & WS
ZMUC-ENP-69	stub	1% OsO4	SEM			12	18.10.2007	32°19.9′N 64°40.6′W	KW & WS
ZMUC-ENP-70	stub	1% OsO4	SEM			12	18.10.2007	32°19.9′N 64°40.6′W	KW & WS
ZMUC-ENP-71	stub	1% OsO4	SEM			12	18.10.2007	32°19.9′N 64°40.6′W	KW & WS
ZMUC-ENP-72	stub	1% OsO4	SEM			12	18.10.2007	32°19.9′N 64°40.6′W	KW & WS
ZMUC-ENP-73	PFA (ca. 4 specimens)	PFA	none			12	18.10.2007	32°19.9′N 64°40.6′W	KW & WS
ZMUC-ENP-74	PBS+NaN_3_ (ca. 7 specimens)	PFA	none			12	18.10.2007	32°19.9′N 64°40.6′W	KW & WS
ZMUC-ENP-75	PBS+NaN3 (ca. 5specimens)	PFA	none			12	18.10.2007	32°19.9′N 64°40.6′W	KW & WS
ZMUC-ENP-76	Caco- buffer (10 specimens)	GLU	none			12	18.10.2007	32°19.9′N 64°40.6′W	KW & WS
ZMUC-ENP-77	Caco- buffer (7 specimens)	GLU	none			12	18.10.2007	32°19.9′N 64°40.6′W	KW & WS
ZMUC-ENP-78	Caco-buffer (5 specimens)	GLU	none			12	18.10.2007	32°19.9′N 64°40.6′W	KW & WS
ZMUC-ENP-79	96% etoh (1 specimen)	96% etoh	none			2–4	17.10.2007	32°19.95′N, 64°43.69′W	KW & WS
ZMUC-ENP-80	96% etoh (5 specimens)	96% etoh	none			12	18.10.2007	32°19.9′N 64°40.6′W	KW & WS
ZMUC-ENP-81	96% etoh (1 specimens)	96% etoh	none			12	18.10.2007	32°19.9′N 64°40.6′W	KW & WS
ZMUC-ENP-82	96% etoh (1 specimens)	96% etoh	none			12	18.10.2007	32°19.9′N 64°40.6′W	KW & WS
ZMUC-ENP-83	96% etoh (1 specimens)	96% etoh	none			12	18.10.2007	32°19.9′N 64°40.6′W	KW & WS

Abbreviations: α-tub, α-tubulin immunostaining (IS); CLSM, confocal laser scanning microscopy; FMRF, FMRF-amid-like IS; GLU, 2–3% glutaraldehyde; Hist, histological semithin sections examined in LM; KW, Katrine Worsaae; LM, light microcopy; PFA, 2–3% paraformaldehyde; phal, phalloidin; SEM, scanning electron microscopy; ser, serotonin IS; TEM, transmission electron microcopy; tyr-tub, tyrosinated tubulin IS; TB, toluidin blue staining of semithin sections on slide; UA+LC, uranyl-acetate+lead citrate staining of ultrathin sections on grid; WM, permanent whole mount (sealed, glycerol); WS, Wolfgang Sterrer.

### Description

*Measurements on holotype; ranges in parenthesis including paratypes and live recordings (see* *Table 1* *).*

**Figure 2:**
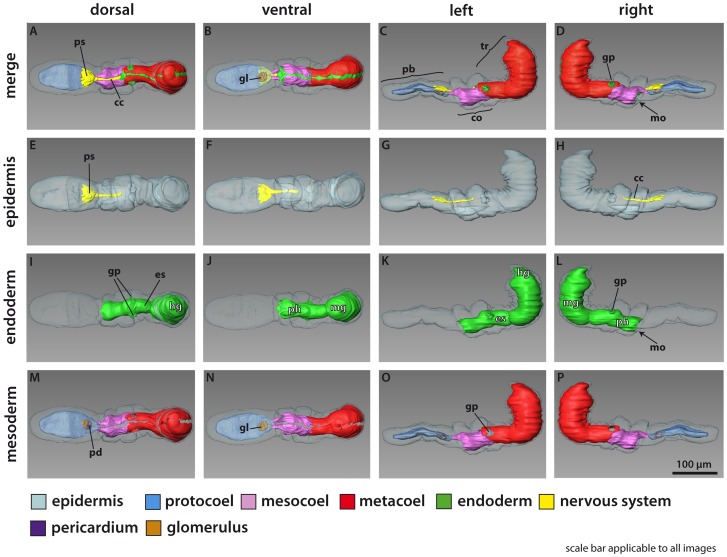
*Meioglossus psammophilus* gen. et sp. nov., 3D-reconstruction of the anatomy of the main organ systems. Rows from left to right: dorsal, ventral, left and right view. Columns from top to bottom: The merge row (A–D) shows the adult with all reconstructed structures. Note that blood vessels and septa separating the paired coelomic cavities are not shown. Epidermis row (E–H) shows the external shape of *M. psammophilus* and condensed nerve structures including the collar cord and proboscis stem. Endoderm row (I–L): The digestive tract is subdivided into five regions, i.e. mouth opening, pharynx, esophagus, midgut and hindgut. Anus not shown. Mesoderm row (M–P) shows the position of the anterior protocoel (blue) and the paired meso- (pink) and metacoelic (red) compartments. Perihaemal diverticula not shown. The glomerulus is visible and occupied at least a quarter of the protocoelomic cavity. The pericardium is situated dorsal to the glomerulus. Click on image in Figure S2 to activate 3D model (follow link to full interactive PDF version of Figure 2). Abbreviations: cc, collar cord; co, collar region; es, esophagus; gl, glomerulus; gp, gill pore; hg, hindgut; mg, midgut; mo, mouth opening; pb, proboscis; pd, pericardium; ph, pharynx; ps, proboscis stem; tr, trunk.

**Figure 3:**
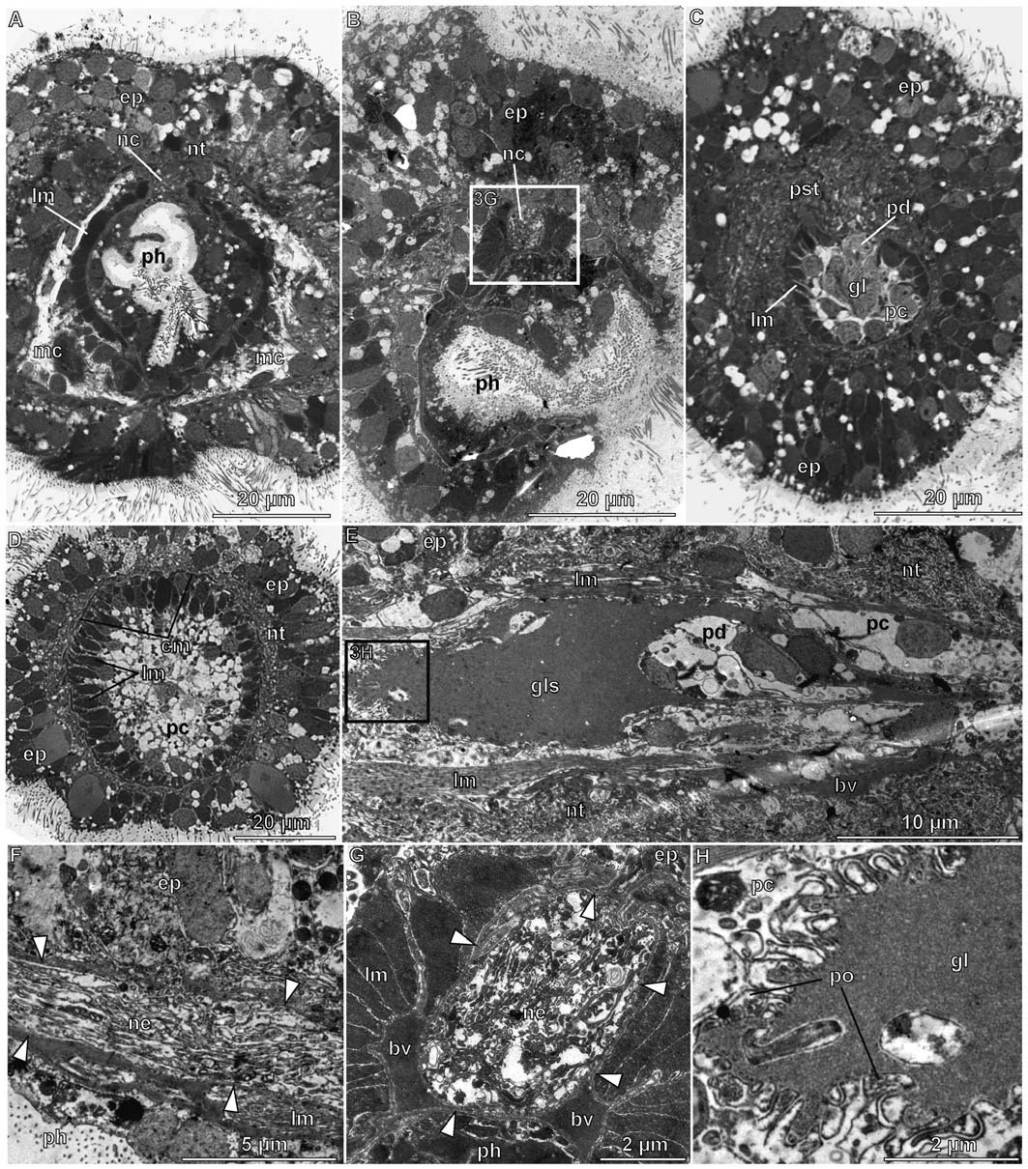
*Meioglossus psammophilus* gen. et sp. nov., light- and transmission electron micrographs of cross sections (A–D, G), and transmission electron micrographs of sagittal sections (E, F, H). A. Cross section of posterior pharynx region. B. Oblique cross section of anterior pharynx region showing mouth opening at the bottom right. No stomochord or proboscis skeleton is present. C. Cross section of proboscis region showing glomerulus and pericardium. D. Cross section of anterior proboscis region showing the arrangement of the muscle fibers. E. Sagittal section of pericardium-glomerulus complex, anterior is to the left. F, G close-ups of the subepidermal collar cord that is constituted of numerous neurites. H. The glomerulus is lined by podocytes from the protocoelic side. Abbreviations: arrowheads, extracellular matrix; bv, blood vessel; cm, circular muscle fibers; ep, epidermis; gl, glomerulus; gls, glomerular sinus; lm, longitudinal muscle fibers; mc, mesocoel; nc, neurocord; ne, neurites; nt, nerve tissue; pc, protocoel; pd, pericardium; ph, pharynx; po, podocytes; pst, proboscis stem.

**Figure 4:**
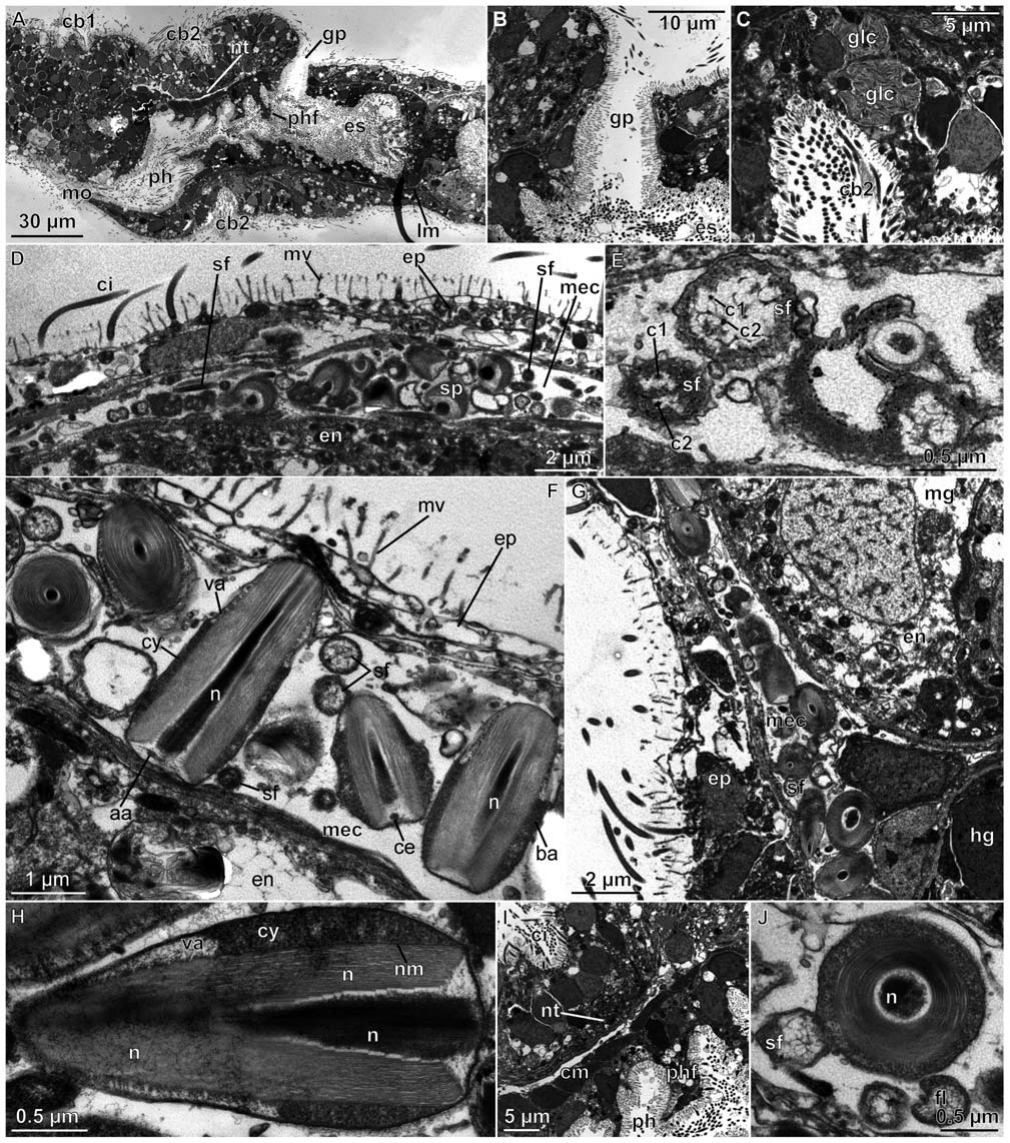
*Meioglossus psammophilus* gen. et sp. nov., sagittal sections, transmission electron micrographs. A. Median section of collar region, showing pharyngeal lumen and esophagus. B. Close up of gill pore. C. Close up of ventral side showing middle groove and ciliary band lined by glandular cells. D. Dorsal trunk, epidermis, sperm and flagella. E. Sperm flagella (double cilia in left flagellum?). F. Close up of sperm, transverse and sagittal view. G. Flagella and sperm heads in metacoel. H. Sperm head, sagittal, median view. I. Close up from A of dorsal pharyngeal foldings and basiepidermal nerve tissue. J. Flagellae and sperm head, transverse view. Abbreviations: aa, anchoring fibre apparatus; ba, electron dense cytoplasma basally; c1, c2, double cilia of flagella; cy, cytoplasma; cb1, cb2, 1. & 2. ciliary band; ce, centriole; ci, cilium; cm, circular muscles; en, endodermal cell; ep, epidermis; es, esophagus; glc, glandular cells; gp, gill pore; hg, hindgut; lm, longitudinal muscles; mec, metacoel; mg, midgut; mo, mouth opening; mv, microvillum; n, nucleus; nt, nerve tissue; nm, nuclear membrane; ph, pharynx; phf, pharyngeal fold; sf, sperm flagellum; sh, sperm head; sp, sperm; va, acrosomal vesicles of anterior cytoplasma.

**Figure 5:**
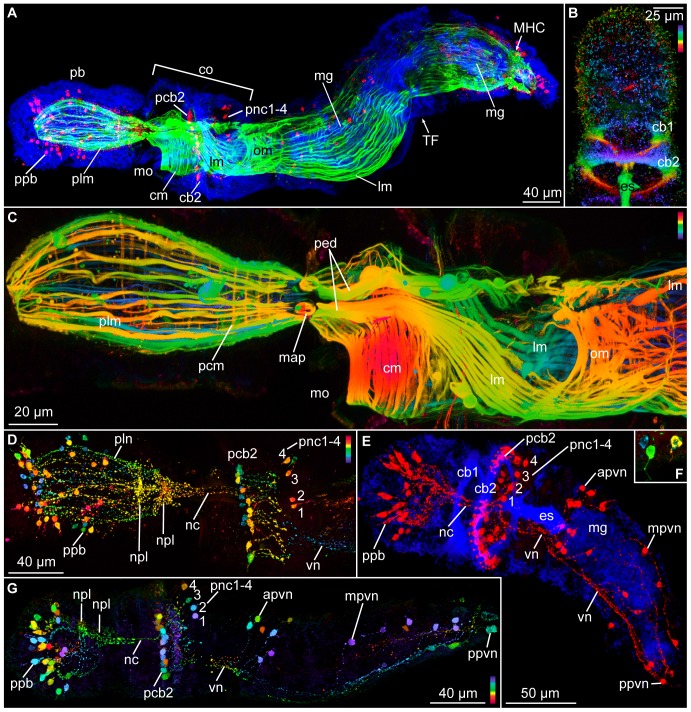
*Meioglossus psammophilus* gen. et sp. nov., confocal laser scanning microscopy, Z-stack projection images showing musculature (phalloidin staining), cilia (anti acetylated α-tubulin IR), nerves (anti α-tubulin and serotonin IR). A. Two zooids of specimen showing paratomy, paratype ZMUC-ENP-3, dorso-lateral view. Triple staining of cilia, muscles and nervous system showing F-actin (green) and anti acetylated α-tubulin IR (blue) and anti-serotonin IR (red). B. Proboscis and collar cilia, paratype ZMUC-ENP-43, anti acetylated α-tubulin IR, depth coded. Depth scale follows the colors of the spectral light (blue to red). C. Anterior part of specimen from A, musculature, depth coded Z-stack. D. Proboscis, serotonergic nervous system (SNS) of A, depth coded Z-stack. E. Whole single specimen, ZMUC-ENP-47, double staining, anti acetylated α-tubulin IR (blue) and anti serotonin IR (red). Same specimen used in 3D isosurface reconstruction in attached Movie S3. F. Close up of serotonergic perikarya from proboscis of G, depth coded. G. Whole specimen, ZMUC-ENP-47, SNS, depth coded. Abbreviations: apvn, anterior perikarya of ventral nerve net; cb1, cb2, 1. & 2. ciliary band; cm, circular muscles; co, collar region; es, esophagus; lm, longitudinal muscles; map, muscular anchoring point; mg, midgut; MHC, midgut-hindgut constriction; mo, mouth opening; mpvn, median perikarya of ventral nerve net; nc, neurochord (collar); npl, nerve plexus; om, oblique muscles; pb, proboscis; pcb2, perikarya of second ciliary band nerves; pcm, proboscis circular muscles; ped, perihaemal diverticula; pnc1–4, posterior perikarya 1 to 4 of neurochord; plm, proboscis longitudinal muscles; pln, nerves innervating proboscis longitudinal muscles; ppb, perikarya of proboscis; ppvn, posterior perikarya of ventral nerve net; TF, transverse fission zone; vn, ventral nerve net (forms two ventral ‘cords’).

**Figure 6:**
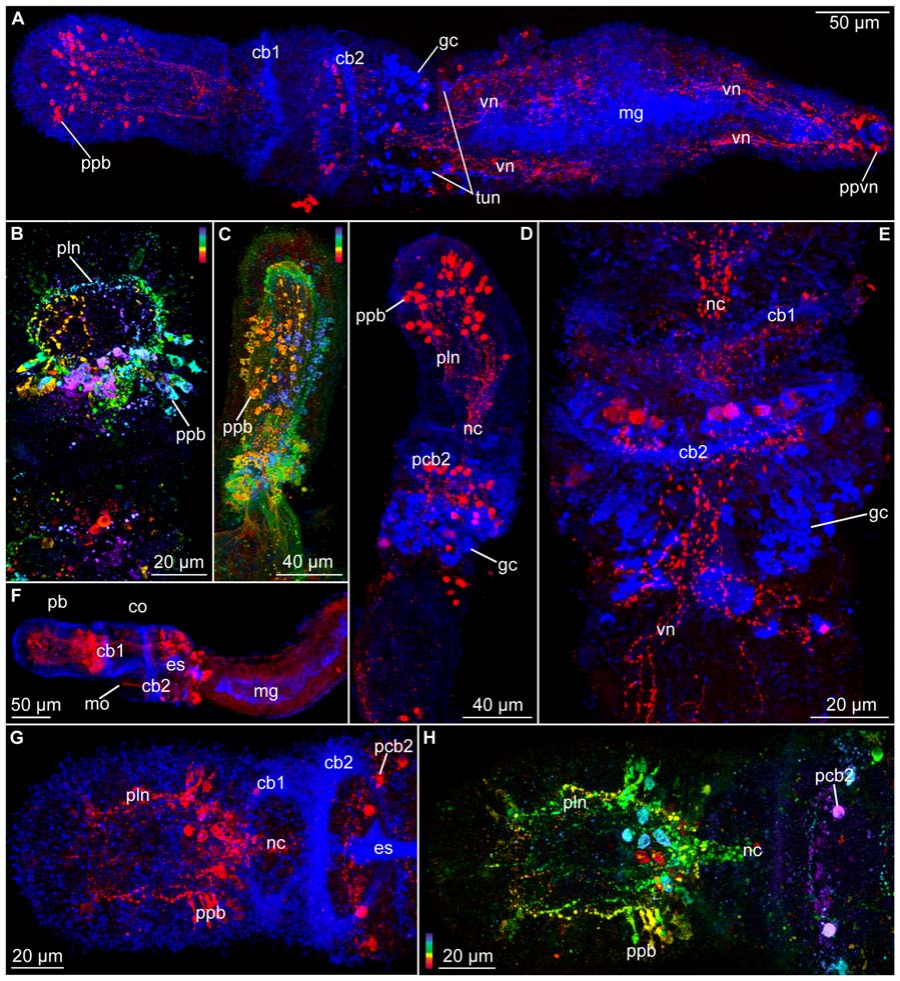
*Meioglossus psammophilus* gen. et sp. nov., confocal laser scanning microscopy, Z-stack projection images showing cilia or glands (anti acetylated α-tubulin IR or tyrosinated tubulin IR) and nerves (anti serotonin- and FMRFamide-like IR). A. Whole specimen, holotype ZMUC-ENP-1, ventral view, double staining of cilia and nervous system showing anti-tyrosinated tubulin IR (blue) and anti serotonin IR (red). B. Proboscis and collar of ZMUC-ENP-49, dorsal view, anti FMRFamide-like IR, depth coded. Depth scale follows the colors of the spectral light (blue to red). C. Proboscis of paratype ZMUC-ENP-5, lateral view, anti FMRFamide-like IR, depth coded Z-stack projection. D. Anterior, dorsal view of ZMUC-ENP-51, anti tyrosinated tubulin IR (blue) and anti serotonin IR (red). E. Collar region of ZMUC-ENP-52, dorsal view, anti tyrosinated tubulin IR (blue) and anti serotonin IR (red). F. Anterior end of paratype ZMUC-ENP-4, lateral view, anti-acetylated α-tubulin IR (blue) and anti FMRFamide-like IR (red). G–H. Proboscis and collar of ZMUC-ENP-50 dorsal view; G. Anti acetylated α-tubulin IR (blue) and anti FMRFamide-like IR (red); H. Anti FMRFamide-like IR, depth coded Z-stack. Abbreviations: apvn, anterior perikarya of ventral nerve net; cb1, cb2, 1. & 2. ciliary band; co, collar region; es, esophagus; gc, glandular cell; mg, midgut; mo, mouth opening; mpvn, median perikarya of ventral nerve net; nc, neurochord (collar); pb, proboscis; pcb2, perikarya of second ciliary band nerves; pln, nerves innervating proboscis longitudinal muscles; ppb, perikarya of proboscis; ppvn, posterior perikarya of ventral nerve net; tun, tubulinergic nerve; vn, ventral nerve net (forms two ventral ‘cords’).

Body slender, contractile, 611 µm long (260–611 µm). Proboscis 170 µm long (92–178 µm) and 89 µm wide (50–91 µm), 1/3 the length of animal when stretched; no mid-dorsal groove; collar 115 µm long (62–150 µm) and 115 µm wide (63–115 µm), trunk 326 µm long (130–326 µm) and 115 µm wide (56–115 µm) (Figures 1A, C–G, 2). Proboscis neck indistinctive; only a shallow constriction between proboscis and collar region. Instead, posterior and lateral areas of proboscis epidermis are connected directly to collar epidermis; ventral mouth opening area restricted (Figures 1A, B, D, 2). Neck supporting structures such as stomochord and nuchal skeleton not present (Figure 3B, C). Epidermis composed of single layer of mucus-secreting, multiciliated cells, with two circumferential ciliary bands. Anterior band lining posterior proboscis border (pre-oral ciliary organ) and terminating in v-shape ventrally in front of the mouth opening (Figure 1A). Posterior ciliary band lining circumbuccal groove that subdivides the collar region into an anterior and posterior ridge (Figures 1A, 4A, C, 5A, B, E, 6A, E–G). Single pair of simple gill pores, gill bars absent. Locomotion slow, primarily ciliary though guided by the muscular proboscis and by a reversal in ciliary beat, equally efficient in anterior as well as posterior direction (see online movies of live animals). Hepatic sacculation and gonadal wings lacking (Figure 1A–G).

Proboscis with protocoel as narrow, unpaired, central lumen extending three quarters the length (Figures 1A, D, 2) and with paired posterior dorso-lateral proboscis pores; pores only detected through 3D reconstruction of serial semi-thick sections (Figure 2, activate 3D model and see view B). Protocoel housing the pericardium-glomerulus complex (Figure 3C, E). Pericardium as small coelomic sac (ca. 8 µm long, 8 µm wide, 7 µm high); ventrally adjoined by voluminous glomerular vessel and postero-dorsally attached to the extracellular matrix (ECM) separating protocoel and epidermis. Glomerulus as enlarged blood plexus within ECM (about 27 µm in length, 23 µm in width and 13 µm in height), lined by protocoelic cells (podocytes, supposedly mediating ultrafiltration) (Figure 3E, H). Pericardium-glomerulus complex attached to posterior wall of protocoel only by strands of ECM, also containing longitudinal blood vessel from posterior body region (Figures 2, view C in activated 3D model; 3E); stomochord absent. Paired mesocoels present along collar region, separated dorsally and ventrally by longitudinal septa (Figure 3A), and posteriorly separated from metacoels by transverse septum, located anterior of trunk gill pores at the anterior trunk (Figure 2). Mesocoelic cavity narrow to indistinct, mesocoelic canals absent. Paired metacoels (separated by dorsal and ventral longitudinal septa) extend along entire trunk region (Figures 1B, 2); metacoels dorso-anteriorly projecting as perihaemal cavities through collar region into base of the proboscis (neck absent) with anterior-most position recognized only by presence of paired dorsal longitudinal muscle bundles (Figure 5C); ventro-lateral peribuccal cavities absent. Large endodermal cells of midgut severely reduce coelomic space of trunk metacoels (Figure 1A, C, D).

Mouth opens ventrally on anterior margin of collar into short but wide buccal cavity, followed by collar and pharyngeal region of the trunk. When specimen is retracted, transverse folds protrude into the pharyngeal lumen (Figures 1A, C–D, 3A, B, 4A, I). A single pair of indistinct dorso-lateral gill pores (best detected with sectioning) present on anterior margin of trunk (Figures 1D, 2, 4A–B). Entire gill duct and circular gill pore lined by non-ciliated cells with cell surfaces bordered evenly by slender microvilli measuring about 2 µm in length (Figure 4A, B). Primary and secondary gill bars absent. Pharynx followed by esophagus posterior of the gill pores within anterior trunk region (Figures 1A–D, 3A, B, 4A); esophagus delineated posteriorly by a muscular constriction before the midgut and heavily ciliated hindgut (Figures 1A, C–D, 4A, 5A, C). Dorsal supraterminal anus followed by a short postanal tail containing numerous 10 µm long oval glandules (8–14 µm) (Figure 1C). In several individuals the midgut contained diatoms (see online LM movie of live animal).

Body wall musculature composed of simple smooth inner longitudinal muscles, extending the full body length, and outer circular muscles, most developed in proboscis (locomotory organ) and lacking in posterior trunk (Figures 3C–E, 5A, C). Besides, inner circular and longitudinal muscles surrounds the intestinal system; with ‘sphincter’ muscles at esophagus-midgut (Figure 5A, C) and midgut-hindgut borders (Figure 1C, 5A); anal sphincter not detected. The proboscis body wall contains longitudinal muscles lining the protocoel with a single ring of about 40–60 muscle fibers (1–3 µm thick, up to 10 µm apart); external to these are about 30 circular muscles (1–2 µm thick, 3–6 µm apart) forming an orthogonal muscle grid together with the longitudinal muscles (Figures 1D, 3C, D, 5A, C; see online CLSM 3D movie of proboscis). The collar contains about 20 dense circular muscles and about 10 thinner slightly oblique circular muscles only visible at the dorsal part of collar, with all 30 collar muscles lining the paired mesocoels (Figures 4I, 5A, C). Two round and ca. 10 µm long phalloidin-stained, presumably muscular structures of unknown function were detected at base of proboscis (map), where proboscis muscles meet trunk muscles, and near the points where proboscis pores connect to protocoel. The two dorsal muscle bundles of each 10 longitudinal trunk strands terminate at map (Figure 5C), both extending antero-dorsally from the trunk as perihaemal diverticula (extending metacoels) into the collar. Along the trunk in posterior direction, these paired muscle bundles run first ventrally along the anterior esophagus, except for a few splitting off dorsally, and a few additional oblique running laterally (Figure 5C; see online CLSM 3D movie of trunk). Along the mid-esophagus, additional longitudinal, oblique and diagonal muscles join the two bundles; all of them fanning out along the posterior esophagus to form a dense net surrounding the trunk, only interrupted by the dorsal and ventral longitudinal septa. The net contains about 40 longitudinal muscles (1–3 µm thick, evenly distributed), continuing along the posterior trunk to insert at the posterior hindgut (Figure 5A, C). No external body wall circular muscles were found in the posterior trunk region.

The enteropneust nervous system is mainly composed of neuroepithelial nerve cells with cell bodies embedded in the epidermis and neurites forming a basiepidermal nerve plexus. In *M. psammophilus* gen. et sp. nov. the nerve net is most distinctive in the proboscis, in a circumferential nerve ring around the collar region, and within the trunk region as a longitudinal mid-ventral condensation beneath the esophagus continuing posteriorly into two main nerve strands (Figures 3A–G, 5D, E, G, 6; online 3D movie of anti-serotonin IR). At the level of the collar region the nervous system constitutes the typical collar cord that lies in a subepidermal position running within the dorsal septum of the mesocoelic cavity, thereby passing through the mesocoelic cavity within the anterior ridge of the collar (Figures 2, 3A, B, F, G). The collar cord (50 µm in total length, 3 µm wide, 5 µm high) is surrounded by ECM and composed of numerous neurites passing the cord in longitudinal direction (Figure 3F); no neuronal somata present within collar cord (Figure 3F, G, 5D, E, G). The collar cord continues into a basiepidermal nerve plexus within the posterior ridge of collar region (Figure 2, 3A, 5D, G, see also online 3D movie of anti-serotonin IR); neuropores absent. Anteriorly, the collar cord connects to the dorsal proboscis stem (Figure 2), showing a condensed area of basiepidermal neurites (Figures 3C, 5D–G, 6A, B–E). Serotonin-like and anti-FMRF-amide like immunoreactivity (IR) was found throughout the nervous system, the serotonergic nervous system being the most distinct. Tubulin IR in nerves could only occasionally be distinguished from IR in cilia (e.g., Figure 6A), and is not described herein. Basiepidermal serotonergic and FMRFamidergic neurites run along the proboscis showing a condensation (npl) in the nerve net at the base of the proboscis, i.e. the proboscis stem (Figures 5D, E, G, 6A–D, G–H). The serotonergic neurites seemingly originate at a dense anterior assemblage of about 30 serotonergic perikarya (Figures 5A, D, E, G, 6A, D) and a median ring of about 20 serotonergic perikarya (Figures 5A, D, G, 6D). The latter plexus also shows 50+ FMRFamidergic perikarya (Figures 6B–C, G–H). Several neurons are distally ciliated (Figure 5F) indicating that these may function as sensory neurons, whereas others may be secretory or motor neurons innervating the proboscis musculature. At the posterior border of the collar four seemingly sensory serotonergic perikarya connected to cilia (pnc, Figure 5A, D–E, G; see also online 3D movie of specimen shown in Figure 5E) give rise to four neurites, fusing anteriorly with the collar cord. The collar cord shows a dense serotonergic and FMRFamidergic IR throughout its entire length along the collar to the base of the proboscis. At the mid-collar a ring of 20–30 serotonergic and FMRFamidergic perikarya are found beneath the dense ciliary band lining the circumbuccal groove (pcb2, Figures 5A, D–E, G, 6D, G–H). Posterior to this ciliary band in the anterior trunk (surrounding the esophagus) dense aggregations of glandular cells are revealed by the presence of tyrosinated tubulin IR (Figures 6A, D, E). The serotonergic basiepidermal nerve net of the anterior trunk is condensed into a mid-ventral cord beneath the esophagus, separating into two ventral bundles at the anterior border of the gut. Each bundle consists of about ten serotonergic neurons with peripheral perikarya embedded in the epithelium along the postero-lateral side, and neurites forming a net around the posterior trunk, possibly innervating the longitudinal musculature (Figures 5D–E, G, 6A, E; see also online 3D movie of specimen shown in Figure 5E). The serotonergic perikarya and associated neurites of the two ventral trunk bundles are organized in pairs along the dorso-lateral side of trunk. The antero-most pair consists actually of two clusters, each comprising 6–7 serotonergic perikarya. The following 4–5 pairs consist of a single serotonergic perikaryon on each side, the last pair reaching the tail (Figure 5E, G).

Reproduction is presumably gonochoristic as in other enteropneusts, but only males were found. Reproduction seemingly seasonal with fertile males found in Belize in January and April (nearly all specimens examined were fertile) and in Bermuda in June but not in October. Sperm distributed in up to four pairs of dorsal testes or freely in the metacoel along the entire intestine; also present in the posterior zooid, during paratomy of fertile animals (Figures 1A–D, H–J, 4D–H, J; see also online LM movie of live animal). Sperm head arrow- or bullet-shaped (2–3 µm long, 0.5–1 µm wide; Figures 1H–J, 4D, F–H, J). Central nucleus with electron-dense core and less dense periphery, sometimes with ‘lamellar’ appearance possibly due to crystallization (Figure 4F, G, H, J). Nucleus surrounded by indistinct nuclear membrane (nm, Figure 4H) and cytoplasma (cy); dense basally (ba) and anteriorly with acrosomal vesicles (va, Figure 4F, H). No mitochondria detected. Centriole and anchoring apparatus (Figure 4F) basal to what appears to be the very long, stiff, possibly degenerated or undeveloped flagellum (up to 30 µm long, 0.5 µm wide), since no microtubules were identified (Figures 1I, 4D–H, J).

A mode of asexual reproduction unique among Enteropneusta was observed in specimens from all collecting sites, during all seasons (in up to one third of the specimens per site). Fertile as well as unfertile individuals divided by paratomy. Paratomy is a form of transverse fission, where the secondary individual develops to almost full size following the anterior-posterior axis in a chain of zooids, before fission (Figures 1C, E, G, 5A). The intestinal system continues through the second zooid (Figures 1C, 5A), seemingly functioning and providing nutrition to the second zooid until shortly before separation (Figure 1G), where the organ differentiation (‘regeneration’) is nearly complete.

**Figure 7:**
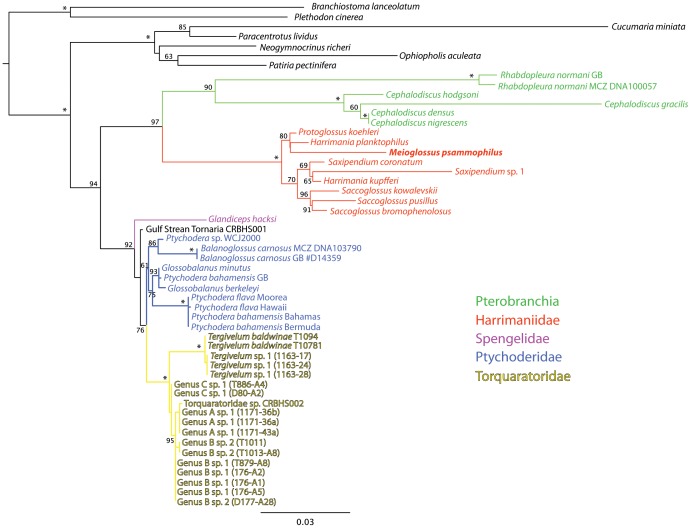
Optimal maximum likelihood tree obtained in RaxML under GTR+Γ for the 18S rRNA data set (−*log*L = −8584.713814). Values on nodes indicate bootstrap support values above 50%; an asterisk indicates a value of 100%.

**Figure 8:**
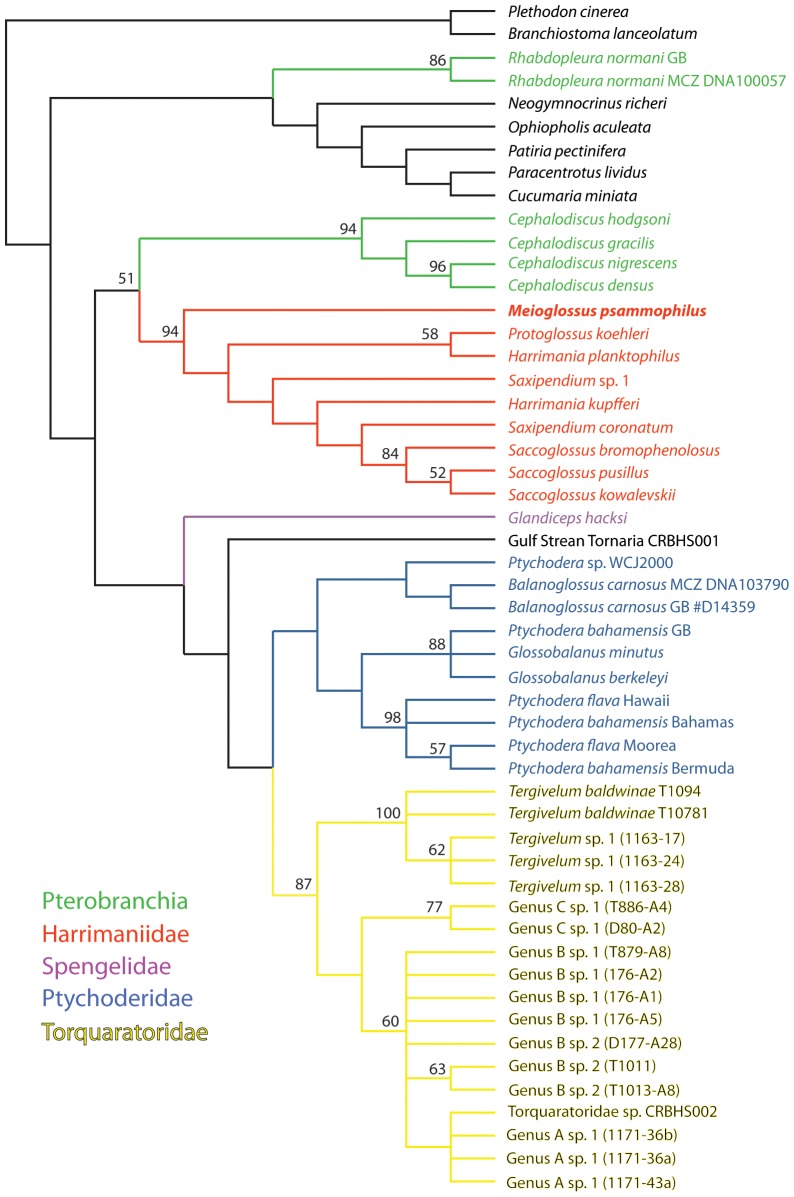
Optimal tree at 4456 weighted steps based on the analysis of 18S rRNA under direct optimization in POY (parameter set 3221). Values above nodes indicate jackknife support values above 50%.

The complete 18S rRNA and a fragment of the mitochondrial 16S rRNA was sequenced from a specimen from Windsor Beach, Bermuda (GenBank Accession numbers in Table 2). Phylogenetic analyses based on 18S rRNA and 16S rRNA showed no identity of *M. psammophilus* gen. et sp. nov. to any previously sequenced hemichordate, including *Glossobalanus* and *Ptychodera* (Ptychoderidae), which are the only previously recorded genera from shallow sandy bottoms around Bermuda and the Caribbean Sea. *Meioglossus psammophilus* gen. et sp. nov. forms a clade with the Harrimaniidae genera *Harrimania*, *Protoglossus*, *Saccoglossus* and *Saxipendium* in all analyses (Figures 7 and 8, Figure S1). The exact position of this species received only low nodal support, either nesting within the family as sister to *Harrimania planktophilus* and *Protoglossus koehleri* (in the maximum likelihood analysis, Figure 7) or as sister group to all other harrimaniids (in the parsimony direct optimization analysis, Figure 8).

**Table 2:** Taxon sampling and sequences included in phylogenetic analyses.

		Voucher	18S rRNA	16S rRNA	Publication	
**Cephalodiscidae**	*Cephalodiscus gracilis*		AF236798	-	Cameron et al. 2000	
	*Cephalodiscus hodgsoni*		EU728441	-	Cannon et al. 2009	Antarctica
	*Cephalodiscus nigrescens*		EU728440	-	Cannon et al. 2009	Antarctica
	*Cephalodiscus densus*		EU728439	-	Cannon et al. 2009	Antarctica
**Rhabdopleuridae**	*Rhabdopleura normani*		U15664	-	Halanych 1995	
	***Rhabdopleura normani***	**MCZ DNA100057**	**JF900483-4**	**-**	**This paper**	**Bermuda**
**Ptychoderidae**	*Ptychodera flava*		AF278681	EU728428	Winchell et al. 2001	Hawaii, USA
	*Ptychodera flava*		EU728436	EU728429	Cannon et al. 2009	Moorea
	***Ptychodera bahamensis***	**MCZ DNA101771/4**	**JF900485**	JX855285	**This paper**	**Bahamas**
	***Ptychodera bahamensis***	**MCZ DNA103686**	**JF900486**	**-**	**This paper**	**Bermuda**
	*Ptychodera bahamensis*		AF236802	-	Cameron et al. 2000	
	***Balanoglossus carnosus***	**MCZ DNA103790**	**JF900489**	**-**	**This paper**	**Australia**
	*Balanoglossus carnosus*		D14359	-	Wada & Satoh 1994	
	*Glossobalanus minutus*	MCZ DNA100058	AF119089	-	Giribet et al. 2000	Spain
	*Glossobalanus berkeleyi*		EU728435	EU728426	Cannon et al. 2009	Washington, USA
	*Ptychodera* sp. WCJ2000		AF278685	EU728427	Winchell et al. 2001	Florida, USA
	Gulf Stream Tornaria		EU728437	EU728430	Cannon et al. 2009	Bahamas
**Torquaratoridae**	*Tergivelum baldwinae* (T1094)		EU520509	EU520497	Holland et al. 2009	
	*Tergivelum baldwinae* (T10781)		JN866772	EU520495	Osborn et al. 2012	
	*Tergivelum* sp. 1 (1163-17)		JN886769	JN886752	Osborn et al. 2012	
	*Tergivelum* sp. 1 (1163-24)		JN886770	JN886753	Osborn et al. 2012	
	*Tergivelum* sp. 1 (1163-28)		JN886771	JN886754	Osborn et al. 2012	
	Torquaratoridae sp. CRBHS002		EU728438	EU728431	Cannon et al. 2009	East Pacific Rise
	Genus A sp. 1 (Wide-lipped 1171-36a)		JN886757	JN886740	Osborn et al. 2012	
	Genus A sp. 1 (Wide-lipped 1171-36b)		JN886758	JN886741	Osborn et al. 2012	
	Genus A sp. 1 (Wide-lipped 1174-43a)		JN886759	JN886742	Osborn et al. 2012	
	Genus B sp. 1 (Extra wide-lipped D176-A1)		JN886761	JN886744	Osborn et al. 2012	
	Genus B sp. 1 (Extra wide-lipped D176-A5)		JN886762	JN886745	Osborn et al. 2012	
	Genus B sp. 1 (Extra wide-lipped D176-A2)		JN886763	JN886746	Osborn et al. 2012	
	Genus B sp. 1 (Extra wide-lipped T879-A8)		JN886760	EU520500	Osborn et al. 2012	
	Genus B sp. 2 (Extra wide-lipped T1013-A8)		EU520514	EU520502	Holland et al. 2009	
	Genus B sp. 2 (Extra wide-lipped T1011)		EU520515	EU520503	Holland et al. 2009	
	Genus B sp. 2 (Extra wide-lipped D177-A28)		JN886764	JN886747	Osborn et al. 2012	
	Genus C sp. 1 (Narrow-lipped T886-A4)		EU520511	EU520499	Holland et al. 2009	
	Genus C sp. 1 (Narrow-lipped D80-A2)		JN886768	JN886751	Osborn et al. 2012	
	Genus D sp. 1 (Plain-collared T438)		JN886765	JN886748	Osborn et al. 2012	
	Genus D sp. 1 (Plain-collared D98-pc66)		JN886767	JN886750	Osborn et al. 2012	
	Genus D sp. 2 (Plain-collared 1174-43b)		JN886766	JN886749	Osborn et al. 2012	
**Harrimaniidae**	*Harrimania planktophilus*		AF236799	EU728421	Cameron et al. 2000	British Columbia, Canada
	***Harrimania kupfferi***	**MCZ DNA103688**	**JF900487**	JX855286	**This paper**	**Denmark**
	***Meioglossus psammophilus*** ** gen. et sp. nov.**	**ZMUC-ENP-1**	**JF900488**	JX855287	**This paper**	**Bermuda**
	*Saccoglossus kowalevskii*		L28054	-	Turbeville et al. 1994	
	*Saccoglossus pusillus*		AF236800	EU728422	Cameron et al. 2000	British Columbia, Canada
	*Saccoglossus bromophenolosus*		AF236801	-	Cameron et al. 2000	Washington, USA
	*Protoglossus koehleri*		EU728432	EU728420	Cannon et al. 2009	Australia
	*Saxipendium coronatum*		EU728433	EU728423	Cannon et al. 2009	SE Pacific Rise
	*Saxipendium* sp. 1 (D176-A11)		JN886774	JN886756	Osborn et al. 2012	
**Spengelidae**	*Glandiceps hacksi*		JN886773	JN886755	Osborn et al. 2012	
**VERTEBRATA**	***Plethodon cinerea***		**JF900490**	-	**This paper**	
**CEPHALOCHORDATA**	***Branchiostoma lanceolatum***		**AY428817**	JX855284	**Giribet et al. 2004/This paper**	
**ECHINODERMATA**	*Neogymnocrinus richeri*		AY275895	-	Cohen et al. 2004	
	*Cucumaria miniata*		DQ777082	-	Janies et al. 2011	
	*Paracentrotus lividus*		AY428816	-	Giribet et al. 2004	
	*Patiria pectinifera*		AB084551	-	Matsubara et al.	
	*Ophiopholis aculeata*		DQ060806	-	Janies et al. 2011	

## Remarks

*Meioglossus psammophilus* gen. et sp. nov. shows a closest resemblance morphologically as well as phylogenetically to members of Harrimaniidae. Ptychoderid apomorphies such as the characteristic hepatic sacs or gonadal wings are lacking. Like several members of Harrimaniidae, the new species has two proboscis pores and lacks circular musculature in the posterior trunk [65]. *Meioglossus psammophilus* gen. et sp. nov. resembles *Harrimania kupfferi* von Willemoes-Suhm, 1871 in the presence of elongated, arrow-shaped sperm heads (Figures 1H–J, 4F, H) [66], but the phylogenetic analyses do not support a sister group relationship of the two species. Thus, *Meioglossus* gen. nov. is here described as a new genus, since it differs significantly from adult males of all described harrimaniid genera and does not nest within any harrimaniid genera included in this analyses. It further differs from all known adult harrimaniid genera in its minute adult size, the single thin ring of longitudinal muscles in the proboscis, the absence of stomochord and neck skeleton, and the asexual reproduction by paratomy.

## Discussion

The finding of an enteropneust that remains meiofaunal during its entire life cycle - the smallest acorn worm known to date - highlights the hidden diversity inhabiting the interstitial environment. Its resemblance to juveniles of other species has resulted in this species being overlooked by zoologists [64] and suggests that this or additional species may be found in other localities. *Meioglossus psammophilus* gen. et sp. nov. is also, with its 0.6 mm in maximum size, among the smallest solitary deuterostomes of about 1 mm [22]–[30], only comparable in size to some acoelomorphs.

Whereas various forms of asexual reproduction are found in Enteropneusta (mainly architomy), this is the first example of paratomy. In *M. psammophilus* gen. et sp. nov. organ differentiation takes place before fission (paratomy), leading to aligned chains of zooids (two only) with the development following the anterior-posterior axis of the parental zooid. Asexual budding as an unaligned form of paratomy is found in other deuterostomes, including ophiuroid larvae and colonial pterobranchs [55], [57], [67]. Reproduction by paratomy in *Meioglossus* gen. nov. seems independent of the presence of gametes, as it has been observed in non-fertile individuals from Bermuda (October 2007), and in the sperm-bearing specimens from Belize (January 2010). In the latter case sperm may be utilized as an energy store, as is the case for the ‘regenerates’ (zooids) of the genital region in an asexually reproducing enteropneust [56],[59]. The ultrastructural investigations were mainly carried out on specimens undergoing paratomy, which may also be the reason for the observations of sperm lying freely in the metacoel with seemingly degenerated flagella.

With its extreme small size, short post-anal tail, complete ciliation, single pair of gill pores and lack of neck skeleton, gill bars and mesocoelic canals, *M. psammophilus* gen. et sp. nov. shows a closer similarity to juvenile harrimaniids than to any known adult. It externally resembles the minute early juvenile of e.g., *Harrimania planktophilus*, which lacks a telotroch in the one-gill pore stage [53]. Except for *H. planktophilus* the juveniles of all investigated enteropneusts are significantly larger than the adult *M. psammophilus* gen. et sp. nov e.g., [54], [68], [69]. Furthermore, all studied enteropneust species during the ‘one-gill-pore stage,’ develop a dense ring of long cilia that beat in conspicuous metachronal waves along the rim of the pore e.g., [53], [54], [69]. No synchronously beating cilia exist along the gill pores of *M. psammophilus* gen. et sp. nov. Moreover, the development of gill pores in other enteropneusts are soon followed by a second pair of pores as they increase in length e.g., [53], [54].

Sexual maturity of *M. psammophilus* gen. et sp. nov. is evident for several reasons, the main one being the presence of sperm. Sperm is present in the majority of individuals examined during the reproductive season; repeatedly found at different localities through several collecting trips, with a total of more than one hundred specimens examined over 5 years. In addition, the molecular data do not suggest a match of the new species to any known macrofaunal enteropneust species from Bermuda or the Caribbean. Finally, despite intensive and thorough sampling during the course of years, no larger individuals or any other harrimaniid species have been found in the investigated areas; the macrofauna being exceptionally well known at both the Smithsonian Carrie Bow Cay station, Belize [70] as well as Bermuda [71], the latter possibly being one of the best-known subtropical faunas worldwide. It is also worth noting that the morphology of the studied specimens is similar in all populations (including detailed muscle and nervous system as well as paratomous reproduction) at different locations, and different seasons. An extended distribution of the serotonergic nervous system through the entire trunk, otherwise only extending along the proboscis and collar in adult *Ptychodera flava* [54] may also be indicative of adulthood; yet this hypothesis warrants further studies of additional species (especially harrimaniids). It is also remarkable that the new species exhibits asexual reproduction by transverse fission, only known from adult stages of other enteropneust species with asexual reproduction (yet as architomy rather than paratomy) [56], [58], [59]. The most obvious argument for adulthood of male *M. psammophilus* is possibly the obvious lack of growth during 2 weeks of live observations, whereas enteropneust juveniles of comparable size are known to approximately double their length or more within the same time frame [53], [68], [69].

The finding of males only, despite intensive collecting effort, is however puzzling. Females may remain undiscovered due to a difference in their seasonality to males or a short reproductive season, which is known for e.g., the enteropneust *Ptychodera flava* [54]. Alternatively, the females may live in a different habitat, such as in the water column, at a greater depth, as commensals or parasites on macrofauna, or buried deeper in the sediment. At least six tornaria larvae (including the giant *Planctosphaera*) not matched to their adults are known from the Gulf Stream [72], [73]. Sequential hermaphroditism is known for several marine animals [74], but hermaphroditism has never previously been reported in enteropneusts, yet does exist in Pterobranchia [55]. Dwarf males are known in a number of marine taxa (not in Hemichordata), but are usually quasi-parasites typically cohabiting with females in varying degrees of intimacy [49], [75], [76]. The small number of spermatozoa in *M. psammophilus* gen. et sp. nov. and the poorly developed sperm tails suggest that sperm may be transferred directly to the female or egg mass rather than indirectly spawned into the water column.

Interstitial animals are generally characterized by minute slender forms, unique ciliary patterns, and an often seemingly simple morphology [12], [13]; but see a counter example in the complex morphology of Loricifera [32]. While most macroscopic phyla have meiofaunal representatives in the interstitial environment [13], none had yet been reported for Hemichordata. This is therefore the first adult solitary meiofaunal species in this phylum. From the derived position of *M. psammophilus* gen. et sp. nov. it can be inferred that it evolved by miniaturization. However, it is difficult to determine whether its many simple morphological traits should be interpreted as juvenile (from an arrest of growth due to progenetic origin) or just multiple losses (several uncoordinated evolutionary events) [47], [76]. Yet, taking into consideration the adaptive requirements of the interstitial milieu, favoring macroevolutionary changes [46], [47], [74], as well as the fact that enteropneust juveniles are already pre-adapted to this environment, it is likely that the origin of *M. psammophilus* gen. et sp. nov. is progenetic, as it has been proposed for other meiofaunal deuterostomes [26], [29].

### Hemichordate phylogeny

Our study includes the most comprehensive phylogeny of the phylum Hemichordata and corroborates previous hypotheses of enteropneust paraphyly, as Pterobranchia appears as the sister group of the enteropneust family Harrimaniidae (Figure 7; ML bootstrap proportion (BP) = 97%). This result has found support in earlier studies using 18S rRNA and Bayesian inference [7] or combined 18S and 28S rRNA using maximum likelihood [3], but has been contradicted by a recent study, which finds no support for a sister group relationship of Pterobranchia and Harrimaniidae [8], using a very similar data set to the one we use here. We therefore redid several analyses using alternative alignment methods and trimmings with GBlocks (results not shown), but could not recover their topology lacking support for a Pterobranchia+Harrimaniidae clade using probabilistic methods. However, our parsimony direct optimization analyses failed to recover hemichordate or pterobranch monophyly (Figure 8).

Harrimaniidae, including the genus *Saxipendium* (formerly in the family Saxipendiidae) receives between 94% jackknife support in the POY analyses (Figure 8) and 100% bootstrap support in the ML analyses (Figure 7) (98% for the 16S rRNA data set; Figure S1), and always includes *M. psammophilus*, whose position within the family is largely unresolved—the most-basal member in the POY analyses (Figure 8), but without support for the clade including the remaining species, or more deeply nested in the ML analyses, in one case receiving 80% bootstrap support for a clade that also includes *Protoglossus koehleri* and *Harrimania planktophilus* (Figure 7).

Sister to the Pterobranchia+Harrimaniidae clade is one that includes the members of the families Spengelidae (*Glandiceps hacksi*), the Gulf Stream Tornaria sample [7], and the reciprocally monophyletic families Ptychoderidae and Torquaratoridae, although support for Ptychoderidae is low in our study, and unsupported in the 16S rRNA data set (Figure S1), both probabilistic and parsimony direct optimization analyses recovered very similar topologies (Figures 7–8). Torquaratoridae is likewise well supported in all analyses, and includes considerable diversity, despite their late discovery [5], [77].

## Materials and Methods

### Ethics statement

*Meioglossus psammophilus* gen. et sp. nov. (Hemichordata) were collected during 2005–2010 at shallow depths in coarse coral sand on reefs in Belize and Bermuda, respectively. Collecting of specimens was approved by the Belize Fisheries department, Ministry of agriculture and Fisheries Belize (to Dr. J. Norenburg, Smithsonian GEN/FIS/15/04/09(52)) and the Bermuda Natural History Museum (to W.S.). Type material is deposited at the Natural History Museum, Denmark (SNM) (Table 1).

### Morphological study

Animals were examined alive and fixed for various morphological studies and DNA sequencing following published methods [48], [78] (see Table 1). Live images and video-recordings were made with a Sony HDR-XR520 mounted on a Zeiss AX10 microscope with DIC as well as an Olympus C-5060 mounted on a Leitz Dialux 20 microscope with phase contrast. The musculature and nervous system were investigated with F-actin staining (FITC-labeled or Alexaflour 488 phalloidin, Sigma P-5282 or Invitrogen A12379) and immuno-staining (monoclonal mouse anti-acetylated α-tubulin Sigma T 6793 (CY5-labelled secondary antibody, Jackson Immunoresearch 115-175-062), monoclonal mouse anti-tyrosinated tubulin Sigma T 6793 (CY5-labelled), polyclonal rabbit anti-serotonin Sigma: S5545 (5-HT) and anti-FMRF-amid ImmunoStar: 20091 (FITC- or TRITC-labeled secondary antibodies, Sigma F0382 or F5268) following published protocols [48], [49], [78](Table 1). Stained specimens were examined with a Leica TCS SP5 confocal laser microscope at the Danish Technical University or at SUND (courtesy of M. Givskov & T. Bjarnsholt). Z-stack projections of CLSM images and 3D rendering analyses were performed with Leica LASAF computer software or Imaris® ×64 6.00 (Bitplane AG, Zurich, Switzerland). Computed 2D images were further optimized with Adobe Photoshop CS4 and Adobe Illustrator CS4 for presentation. Specimens were prepared for histological sectioning, transmission electron microscopy and scanning electron microscopy according to published methods [48] (Table 1). Complete longitudinal serial sections of 0.5 µm thickness for light microscopy of three adult specimens (ZMUC-ENP-27 to 29) were sectioned on a Leica Ultracut S; two nearly complete transverse (ZMUC-ENP-30, 34) plus three sagittal series (ZMUC-ENP-31, 32, 33) all based on 0.5–1 µm-thick sections were further sectioned on various Leica ultra-microtomes. All semi-thin sections were stained with toluidine blue. About a thousand longitudinal and transverse thin sections for TEM were cut from six adult specimens on various ultra-microtome models including a Leica Ultracut 7 (se Table 1). Ultrathin sections were stained with 2% uranylacetate and 2.5% lead citrate and examined with a JEM-1010 at BIO, NATFAK and Philips 300 at SUND, University of Copenhagen and a ZEISS 902 at CIUS, University of Vienna. Light microscopic images of semithin sections were recorded with a digital camera mounted on a Nikon Eclipse E800 compound microscope or with a DP71 digital camera mounted on an Olympus BX50 compound microscope. Light microscopic images from a complete longitudinal series were aligned using open source software Imodalign on a Linux operating computer [79]. Based on the resulting stack of images a 3D-model of the anatomy of the main organ systems was created in Amira 5.4.1 software (Visage Imaging, Berlin, Germany). Scanning electron images of critical point dried and platinum coated specimens were taken with a JEOL JSM-6335F Field Emission scanning electron microscope at SNM, NATFAK, University of Copenhagen.

### Molecular study

Genomic DNA was isolated from *M. psammophilus* gen. et sp. nov. from Bermuda using the DNeasy Tissue Kit (Qiagen) and the complete 18S rRNA and a fragment of the mitochondrial 16S rRNA were sequenced (GenBank accession numbers in Table 2). Although we attempted to sequence other specimens from Bermuda and Belize, only one yielded good quality DNA, and amplified for multiple markers. Another specimen amplified for only a short gene, histone H3.

A total of 52 18S rRNA sequences were obtained from previous studies [5]–[8] or were sequenced for this study to span the hemichordate diversity. The sampling includes members from all described families, and a large diversity of the recently discovered Torquaratoridae [5], [77]. We only used nearly-complete 18S rRNA sequences, with the exception of rhabdopleurids, for which no complete 18S rRNA sequences were available. Sequencing 18S rRNA followed standard protocols and primers described elsewhere [80]. Primers and 5′ and 3′ edges were trimmed to accommodate partial sequences from some of the studies listed above, for a total unaligned length of between 1707 and 1831 nucleotides. We avoided the sequence of *Stereobalanus canadensis* (GenBank # EU728434) as it is of questionable origin. Likewise we obtained 36 16S rRNA sequences, but no 16S rRNA is currently available for any members of Pterobranchia, and therefore the 16S rRNA data set has no bearing in the debate of enteropneust monophyly. For this reason, we analyzed it independently, with the sole aim of testing the affinity of *M. psammophilus* gen. et sp. nov.

### Phylogenetic analyses - Probabilistic approaches

Maximum likelihood (ML) analyses of the 18S rRNA data set were conducted on a static alignment generated by MUSCLE v. 3.6 [81] with default parameters, yielding 2034 aligned positions, which were reduced to 1570 positions after treatment with Gblocks 0.91b [82]. The same treatment was given to the 16S rRNA data set, which included 603 aligned positions, from which 510 were retained after treatment with Gblocks. Each individual data set was then submitted to ML analysis using RAxML v. 7.2.7 [83] through the CIPRES v. 3 gateway, using 64 processors in a cluster housed at the National Center for Supercomputing Applications (University of Illinois). A unique GTR model of sequence evolution with corrections for a discrete gamma distribution (GTR+Γ) was specified for the data. Nodal support was estimated via the rapid bootstrap algorithm (1000 replicates) using the GTR-CAT model [84].

### Phylogenetic analyses - Dynamic homology under parsimony

A direct optimization [85] analysis for the 18S rRNA was conducted in POY v. 4 [86] under parameter set 3221 (indel opening = 3; indel extension = 1; transversions = transitions = 2) using a timed search with TBR branch swapping, tree fusing and ratcheting, searching for 6 hours and accumulating all best trees for each replicate. The 33 resulting trees were submitted to a second round of tree fusing. The search evaluated 5 independent repetitions with ratchet and fusing for 7 generations. The shortest tree, at 4456 weighted steps, was found 2 times. Nodal support was evaluated using 1000 jackknife [87] replicates with a probability of deletion of each character of 0.36 (Figure 8). Since resampling techniques may be meaningless under dynamic homology, different strategies can be applied. Dynamic characters can be converted to a static set, but this tends to inflate support values, as it is based on the implied alignment that favors that topology. Instead, we resample dynamic characters by both using the number of predetermined fragments (6 fragments for 18S rRNA) as well as the command *auto_sequence_partition*, which evaluates each predetermined fragment. If a long region appears to have no indels, then the fragment is broken inside that region, effectively increasing the number of characters than can be resampled.

## Supporting Information

Figure S1 **Optimal maximum likelihood tree obtained in RaxML under GTR+Γ for the 16S rRNA data set (−** ***log*** **L = −4667.030968).** Values on nodes indicate bootstrap support values above 50%; an asterisk indicates a value of 100%.
(TIF)

Figure S2 ***Meioglossus psammophilus*** **gen. et sp. nov., 3D-reconstruction of the anatomy of the main organ systems.** Click on image to activate 3D model.
(PDF)

Movie S1 ***Meioglossus psammophilus*** **gen. et sp. nov., recording through dissecting microscope of extracted, live animals in petri dish.**
(MP4)

Movie S2 ***Meioglossus psammophilus*** **gen. et sp. nov., recording through compound microscope of live animal, showing paratomy, ciliary beating, intestinal system, sperm.**
(MP4)

Movie S3 ***Meioglossus psammophilus*** **gen. et sp. nov., confocal laser scanning microscopy of proboscis musculature, 3D rendering of Z-stack performed with Imaris 6.02 software.** Movie showing first all 3 channels of signal with cilia in blue (anti acetylated α-tubulin IR), serotonergic nervous system in red (anti serotonin IR) and musculature in green (phalloidin), thereafter only musculature (green).
(MP4)

Movie S4 ***Meioglossus psammophilus*** **gen. et sp. nov., confocal laser scanning microscopy of collar and anterior trunk musculature, 3D rendering of Z-stack performed with Imaris 6.02 software.** Movie showing first all 3 channels of signal with cilia in blue (anti acetylated α-tubulin IR), serotonergic nervous system in red (anti serotonin IR) and musculature in green (phalloidin), thereafter only musculature (green).
(MP4)

Movie 5 ***Meioglossus psammophilus*** **gen. et sp. nov., confocal laser scanning microscopy, 3D isosurface reconstruction of serotonergic nervous system from Z-stack performed with Imaris 6.02 software.** Movie showing first direct signal of cilia in blue (anti acetylated α-tubulin IR) and serotonergic nervous system in red (anti serotonin IR). Thereafter isosurface manipulation of anti serotonin IR in neurites and perikarya. Neurocord (collar cord) and condensed anterior ventral nerve net colored orange, remaining parts red. Perikarya of proboscis colored green; perikarya 1 to 4 of neurochord on posterior collar colored dark blue; perikarya of second ciliary band nerves colored purple and medium blue; perikarya of ventral nerve net colored light blue.
(MP4)

## References

[1] Maisey JG (1986) Heads and tails: A chordate phylogeny. Cladistics 2: 201–256. 10.1111/j.1096-0031.1986.tb00462.x 34949070

[2] Schaeffer B (1987) Deuterostome monophyly and phylogeny. Evol Biol 21: 179–235.

[3] Winchell CJ, Sullivan J, Cameron CB, Swalla BJ, Mallatt J (2002) Evaluating hypotheses of deuterostome phylogeny and chordate evolution with new LSU and SSU ribosomal DNA data. Mol Biol Evol 19 5: 762–776. 11961109 10.1093/oxfordjournals.molbev.a004134

[4] Cameron CB (2005) A phylogeny of the hemichordates based on morphological characters. Can J Zool 83 1: 196–215.

[5] Holland ND, Clague D, Gordon D, Gebruk A, Pawson D, et al (2005) ‘Lophenteropneust’ hypothesis refuted by collection and photos of new deep-sea hemichordates. Nature 434: 374–376. 15772659 10.1038/nature03382

[6] Cameron CB, Garey JR, Swalla BJ (2000) Evolution of the chordate body plan: New insights from phylogenetic analyses of deuterostome phyla. Proc Nat Acad Sci USA 97: 4469–4474. 10781046 10.1073/pnas.97.9.4469 PMC18258

[7] Cannon JT, Rychel AL, Eccleston H, Halanych KM, Swalla BJ (2009) Molecular phylogeny of Hemichordata, with updated status of deep-sea enteropneusts. Mol Phylogenet Evol 52: 17–24. 19348951 10.1016/j.ympev.2009.03.027

[8] Osborn KJ, Kuhnz LA, Priede IG, Urata M, Gebruk AV, et al (2011) Diversification of acorn worms (Hemichordata, Enteropneusta) revealed in the deep sea. Proc R Soc B: Biol Sci 279: 1646–1654. 10.1098/rspb.2011.1916 PMC3282343 22090391

[9] Benito J, Pardos F (1997) Hemichordata. In: Harrison FW, editor. Microscopic Anatomy of Invertebrates, vol 15. New York: Wiley Liss. pp. 15–101.

[10] Stebbing ARD (1970) Aspects of the reproduction and life cycle of *Rhabdopleura compacta* (Hemichordata). Mar Biol 5: 205–212.

[11] Lester SM (1988) Ultrastructure of adult gonads and development and structure of the larva of *Rhabdopleura normani* (Hemichordata: Pterobranchia). Acta Zool 69 2: 95–109.

[12] Higgins RP, Thiel H (eds)(1988) Introduction to the Study of Meiofauna. Washington: Smithsonian Inst Press. 488 p.

[13] Giere O (2009) Meiobenthology. The microscopic motile fauna of aquatic sediments, 2 edn. Berlin Heidelberg: Springer-Verlag. 527 p.

[14] Ruiz-Trillo I, Riutort M, Littlewood DT, Herniou EA, Baguñà J (1999) Acoel flatworms: earliest extant bilaterian metazoans, not members of Platyhelminthes. Science 283: 1919–1923. 10082465 10.1126/science.283.5409.1919

[15] Ruiz-Trillo I, Riutort M, Fourcade HM, Baguñà J, Boore JL (2004) Mitochondrial genome data support the basal position of Acoelomorpha and the polyphyly of the Platyhelminthes. Mol Phylogenet Evol 33: 321–332. 15336667 10.1016/j.ympev.2004.06.002

[16] Bourlat SJ, Juliusdottir T, Lowe CJ, Freeman R, Aronowicz J, et al (2006) Deuterostome phylogeny reveals monophyletic chordates and the new phylum Xenoturbellida. Nature 444: 85–88. 17051155 10.1038/nature05241

[17] Sempere LF, Martinez P, Cole C, Baguñà J, Peterson KJ (2007) Phylogenetic distribution of microRNAs supports the basal position of acoel flatworms and the polyphyly of Platyhelminthes. Evol Dev 9: 409–415. 17845513 10.1111/j.1525-142X.2007.00180.x

[18] Hejnol A, Obst M, Stamatakis AMO, Rouse GW, Edgecombe GD, et al (2009) Assessing the root of bilaterian animals with scalable phylogenomic methods. Proc R Soc B 276: 4261–4270. 10.1098/rspb.2009.0896 PMC2817096 19759036

[19] Philippe H, Brinkmann H, Copley RR, Moroz LL, Nakano H, et al (2011) Acoelomorph flatworms are deuterostomes related to *Xenoturbella* . Nature 470: 255–258. 21307940 10.1038/nature09676 PMC4025995

[20] Edgecombe G, Giribet G, Dunn CW, Hejnol S, Kristensen RM, et al (2011) Higher-level metazoan relationships: recent progress and remaining questions. Org Div Evol 11: 151–172.

[21] Lowe CJ, Ariel MP (2011) Animal evolution: a soap opera of unremarkable worms. Curr Biol 21: 151–153. 10.1016/j.cub.2010.12.017 21334293

[22] Monniot F (1962) *Dextrogaster suecica* n.g. n.sp. Ascidie interstitielle des graviers du Skagerrak. C R Acad Sci Paris 255: 2820–2822.

[23] Monniot F (1965) Ascidies interstitielles des côtes d'Europe. PhD Thesis, Université de Paris. Mem Mus nat Hist nat Paris 35: 1–154.

[24] Monniot F (1966) Ascidies interstitielles. Veröff Inst Meeresforsch Bremerhaven 161–164.

[25] Rao GC (1968) On *Psammothuria ganapatii* n. gen. n. sp., an interstitial holothurian from the beach sands of waltair coast and its autecology. Proc: Plant Sci 67 5: 201–206.

[26] Swedmark B (1971) A review of Gastropoda, Brachiopoda, and Echinodermata in marine meiobenthos. Smithson Contr Zool 76: 41–45.

[27] Salvini-plawen LV (1972) Die nordatlantische *Labidoplax buskii* (Holothuroidea - Synaptidae) in der Adria. Zool Anz 188: 301–304.

[28] Monniot C, Monniot D, Gaill F (1975) Les Sorberacea: une nouvelle classe de Tuniciers. Arch Zool Exp Gén 116: 77–122.

[29] Monniot C, Monniot F (1978) Recent work on Deep-sea Tunicates. Oceanogr Mar Biol Ann Rev 16: 181–223.

[30] Monniot C, Monniot F (1990) Revision of the class Sorberacea (nethic tunicates) with descriptions of seven new species. Zool J Linn Soc 99 3: 239–290.

[31] Ax P (1956) Die Gnathostomulida, eine rätselhafte Wurmgruppe aus dem Meeressand. Abhandl Akad Wiss u Lit Mainz, math-naturwiss Kl 8: 1–32.

[32] Kristensen RM (1983) Loricifera, a new phylum with Aschelminthes characters from meiobenthos. Z Zool Syst Evolut-forsch 21: 163–180.

[33] Ax P (1996) Multicellular animals: A new approach to the phylogenetic order in nature, Vol. 1. Berlin: Springer-Verlag. 225 p.

[34] Kristensen RM, Funch P (2000) Micrognathozoa: A new class with complicated jaws like those of Rotifera and Gnathostomulida. J Morphol 246: 1–49. 11015715 10.1002/1097-4687(200010)246:1<1::AID-JMOR1>3.0.CO;2-D

[35] Sørensen MV (2002) Further structures in the jaw apparatus of *Limnognathia maerski* (Micrognathozoa) with notes on the phylogeny of the Gnathifera. J Morphol 255: 131–145. 10.1002/jmor.10038 12474262

[36] Willems WR, Curini-Galletti M, Ferrero TJ, Fontaneto D, et al (2009) Meiofauna of the Koster-area, results from a workshop at the Sven Lovén Centre for Marine Sciences (Tjärnö, Sweden). Meiofauna Mar 17: 1–34.

[37] Curini-Galetti M, Artois T, Delogu V, De Smet WH, Fontaneto D, et al (2012) Patterns of diversity in soft-bodied meiofauna: dispersal ability and body size matter. PLoS ONE 7 3: e33801 doi:10.1371/journal.pone.0033801 22457790 10.1371/journal.pone.0033801 PMC3311549

[38] Derycke S, Fonseca G, Vierstraete A, Vanfleteren J, Vincx M, et al (2008) Disentangling taxonomy within the *Rhabditis* (*Pellioditis*) *marina* (Nematoda, Rhabditidae) species complex using molecular and morphological tools. Zool J Linn Soc 152: 1–15.

[39] Casu M, Curini-Galletti M (2006) Genetic evidence for the existence of cryptic species in the mesopsammic flatworm *Pseudomonocelis ophiocephala* (Rhabditophora: Proseriata). Biol J Linn Soc 87: 553–576.

[40] Fontaneto D, Kaya M, Herniou EA, Barraclough TG (2009) Extreme levels of hidden diversity in microscopic animals (Rotifera) revealed by DNA taxonomy. Mol Phylogen Evol 53: 182–189. 10.1016/j.ympev.2009.04.011 19398026

[41] Fontaneto D (ed) (2011) Biogeography of microscopic organisms. Is everything small everywhere? Cambridge: Cambridge University Press. 365 p.

[42] Cooper A, Fortey R (1998) Evolutionary explosions and the phylogenetic fuse. Trends Ecol Evol 13: 151–156. 21238236 10.1016/s0169-5347(97)01277-9

[43] Worsaae K, Kristensen RM (2005) Evolution of interstitial Polychaeta (Annelida). Hydrobiol 535: 319–340.

[44] Sørensen MV, Hebsgaard MB, Heiner I, Glenner H, Willerslev E, et al (2008) New data from an enigmatic phylum: evidence from molecular sequence data supports a sister-group relationship between Loricifera and Nematomorpha. J Zool Syst Evol Res 46: 231–239.

[45] Worsaae K, Nygren A, Rouse GW, Giribet G, Persson J, et al (2005) Phylogenetic position of Nerillidae and *Aberranta* (Polychaeta, Annelida), analysed by direct optimization of combined molecular and morphological data. Zool Scr 34: 313–328.

[46] Rundell RJ, Leander BS (2010) Masters of miniaturization: convergent evolution among interstitial eukaryotes. Bioessays 32: 430–437. 20414901 10.1002/bies.200900116

[47] Westheide W (1987) Progenesis as a principle in meiofauna evolution. J Nat Hist 21: 843–854.

[48] Worsaae K, Rouse GW (2008) Is *Diurodrilus* an annelid? J Morphol 269: 1426–1455. 18985766 10.1002/jmor.10686

[49] Worsaae K, Rouse GW (2010) The simplicity of males: Dwarf males of four species of *Osedax* (Siboglinidae; Annelida) investigated by confocal laser scanning microscopy. J Morphol 271: 127–142. 19658166 10.1002/jmor.10786

[50] Bleidorn C (2007) The role of character loss in phylogenetic reconstruction as exemplified for the Annelida. J Zool Syst Evol Res 45: 299–307.

[51] Struck TH (2006) Progenetic species in polychaetes (Annelida) and problems assessing their phylogenetic affiliation. Integr Comp Biol 46: 558–568. 21672766 10.1093/icb/icj055

[52] Struck TH, Westheide W, Purschke G (2002) Progenesis in Eunicida (“Polychaeta”, Annelida) – separate evolutionary events? Evidence from molecular data. Mol Phylogenet Evol 25: 190–199. 12383760 10.1016/s1055-7903(02)00231-2

[53] Cameron CB (2002) The anatomy, life habits, and later development of a new species of enteropneust, *Harrimania planktophilus* (Hemichordata: Harrimaniidae) from Barkley Sound. Biol Bull 202: 182–191. 11971813 10.2307/1543654

[54] Nielsen C, Hay-Schmidt A (2007) Development of the enteropneust *Ptychodera flava*: Ciliary bands and nervous system. J Morphol 268: 551–570. 17469131 10.1002/jmor.10533

[55] Hadfield MG (1975) Hemichordata. In: Giese AC, Pearse JS, editors. Reproduction of marine invertebrates, Vol II. Entoprocts and lesser coelomates. London: Academic Press. Pp. 185–240.

[56] Petersen JA, Ditadi ASF (1971) Asexual reproduction in *Glossobalanus crozieri* Ptychoderidae, Enteropneusta, Hemichordata). Mar Biol 9: 78–85.

[57] Petersen JA (1994) 10. Hemichordates. In: Adiyodi KG & Adiyodi RG, editors. Reproductive biology of invertebrates. Asexual Propagation and Reproductive Strategies, Vol 6, Part B. Chichester: Wiley. pp. 385–394.

[58] Packard A (1968) Asexual reproduction in *Balanoglossus* (Stomochordata). Proc R Soc Lond B Biol Sci 171: 261–272.

[59] Miyamoto N, Saito Y (2010) Morphological characterization of the asexual reproduction in the acorn worm *Balanoglossus simodensis.* . Dev Growth Diff 52 7: 615–627. 10.1111/j.1440-169X.2010.01197.x 20887562

[60] Kharin AV, Zagainova IV, Kostyuchenko RP (2006) Formation of the paratomic fission zone in freshwater oligochaetes. Russ J Dev Biol 37 6: 354–365. 17168378

[61] Åkesson B, Gschwentner R, Hendelberg J, Ladurner P, Müller J, et al (2001) Fission in *Convolutriloba longifissura*: asexual reproduction in acoelous turbellarians revisited. Acta Zool 82 3: 231–239.

[62] Bernhard E, Gschwentner R, Rieger R (2007) Free-living flatworms under the knife: past and present. Dev Genes Evol 217 2: 89–104. 17146688 10.1007/s00427-006-0120-5 PMC1784541

[63] Dilly PN (1985) The habitat and behaviour of *Cephalodiscus gracilis* (Pterobranchia, Hemichordata) from Bermuda. J Zool Lond 207: 223–239.

[64] Tyler S (2001) The early worm: Origins and relationships of the lower flatworms. In: Bray R, Littlewood DTJ, editors. Interrelationships of the Platyhelminthes. New York: Taylor & Francis. Pp. 3–12.

[65] Deland C, Cameron CB, Rao KP, Ritter WE, Bullock TH (2010) A taxonomic revision of the family Harrimaniidae (Hemichordata: Enteropneusta) with descriptions of seven species from the Eastern Pacific. Zootaxa 2408: 1–30.

[66] Spengel JW (1893) Die Enteropneusten des Golfes von Neapel. Fauna und Flora des Golfes von Neapel 18: 1–757.

[67] Rychel AL, Swalla B J (2009) Regeneration in hemichordates and echinoderms. In: Matranga V, Rinkevich B, editors. Stem cells in marine organisms. Netherlands: Springer. Pp. 245–265.

[68] Kaul S, Stach T (2010) Ontogeny of the collar cord: neurulation in the hemichordate *Saccoglossus kowalevskii* . J Morphol 271: 1240–1259. 20665533 10.1002/jmor.10868

[69] Burdon-Jones C (1952) Development and biology of the larva of *Saccoglossus horsti* (Enteropneusta). Phil Trans R Soc London 639: 553–590.

[70] Rützler K, MacIntyre IG (eds) (1982) The Atlantic Barrier Reef Ecosystem at Carrie Bow Cay, Belize, I: Structure and Communities. Washington: Smiths Inst Pr. 539 p.

[71] Sterrer W (1986) Marine Fauna and Flora of Bermuda. New York: Wiley-Interscience. 742 p.

[72] Garstang W (1939) Spolia Bermudiana. I. On a remarkable new type of *Auricularia* larva (*A. bermudensis*, n. sp.). Quart J Microsc Sci 81: 321–345.

[73] Garstang W (1939) Spolia Bermudiana. II. The ciliary feeding mechanism of *Tornaria* . Quart J Microsc Sci 81: 347–365.

[74] Warner RR (1975) The adaptive significance of sequential hermaphroditism in animals. Amer Nat 109: 61–82.

[75] Vollrath F (1998) Dwarf males. Trends Ecol Evol 13: 159–163. 21238243 10.1016/s0169-5347(97)01283-4

[76] Hanken J, Wake DB (1993) Miniaturization of body size: organismal consequences and evolutionary significance. Ann Rev Ecol Syst 24: 501–19.

[77] Holland ND, Jones WJ, Ellena J, Ruhl HA, Smith KL (2009) A new deep-sea species of epibenthic acorn worm (Hemichordata, Enteropneusta). Zoosystema 31: 333–346.

[78] Nielsen C, Worsaae K (2010) Structure and occurrence of cyphonautes larvae (Bryozoa, Ectoprocta). J Morphol 271: 1094–1109. 20730922 10.1002/jmor.10856

[79] Quast B (2012) High throughput image registration with open source software. 13th annual meeting GfBS 2012, Bonn.

[80] Giribet G, Distel DL, Polz M, Sterrer W, Wheeler WC (2000) Triploblastic relationships with emphasis on the acoelomates and the position of Gnathostomulida, Cycliophora, Plathelminthes, and Chaetognatha: A combined approach of 18S rDNA sequences and morphology. Syst Biol 49: 539–562. 12116426 10.1080/10635159950127385

[81] Edgar RC (2004) MUSCLE: multiple sequence alignment with high accuracy and high throughput. Nucleic Acids Res 32: 1792–1797. 15034147 10.1093/nar/gkh340 PMC390337

[82] Castresana J (2000) Selection of conserved blocks from multiple alignments for their use in phylogenetic analysis. Mol Biol Evol 17: 540–552. 10742046 10.1093/oxfordjournals.molbev.a026334

[83] Stamatakis A (2006) RAxML-VI-HPC: maximum likelihood-based phylogenetic analyses with thousands of taxa and mixed models. Bioinformatics 22: 2688–2690. 16928733 10.1093/bioinformatics/btl446

[84] Stamatakis A, Hoover P, Rougemont J (2008) A rapid bootstrap algorithm for the RAxML Web servers. Syst Biol 57: 758–771. 18853362 10.1080/10635150802429642

[85] Wheeler WC (1996) Optimization alignment: the end of multiple sequence alignment in phylogenetics? Cladistics 12: 1–9.

[86] Varón A, Lucaroni N, Hong L, Wheeler WC (2012) POY version 5.0.0. Software and documentation, American Museum of Natural History, New York. http://research.amnh.org/scicomp.

[87] Farris JS, Albert VA, Källersjö M, Lipscomb D, Kluge AG (1996) Parsimony jackknifing outperforms neighbor-joining. Cladistics 12: 99–124. 10.1111/j.1096-0031.1996.tb00196.x 34920604

