# Supplementary figures and images for: An Anatomical Description of a Miniaturized Acorn Worm (Hemichordata, Enteropneusta) with Asexual Reproduction by Paratomy
Source: PLoS One. 2012 Nov 7;7(11):e48529. doi: 10.1371/journal.pone.0048529 (PMC3492459; doi:10.1371/journal.pone.0048529)

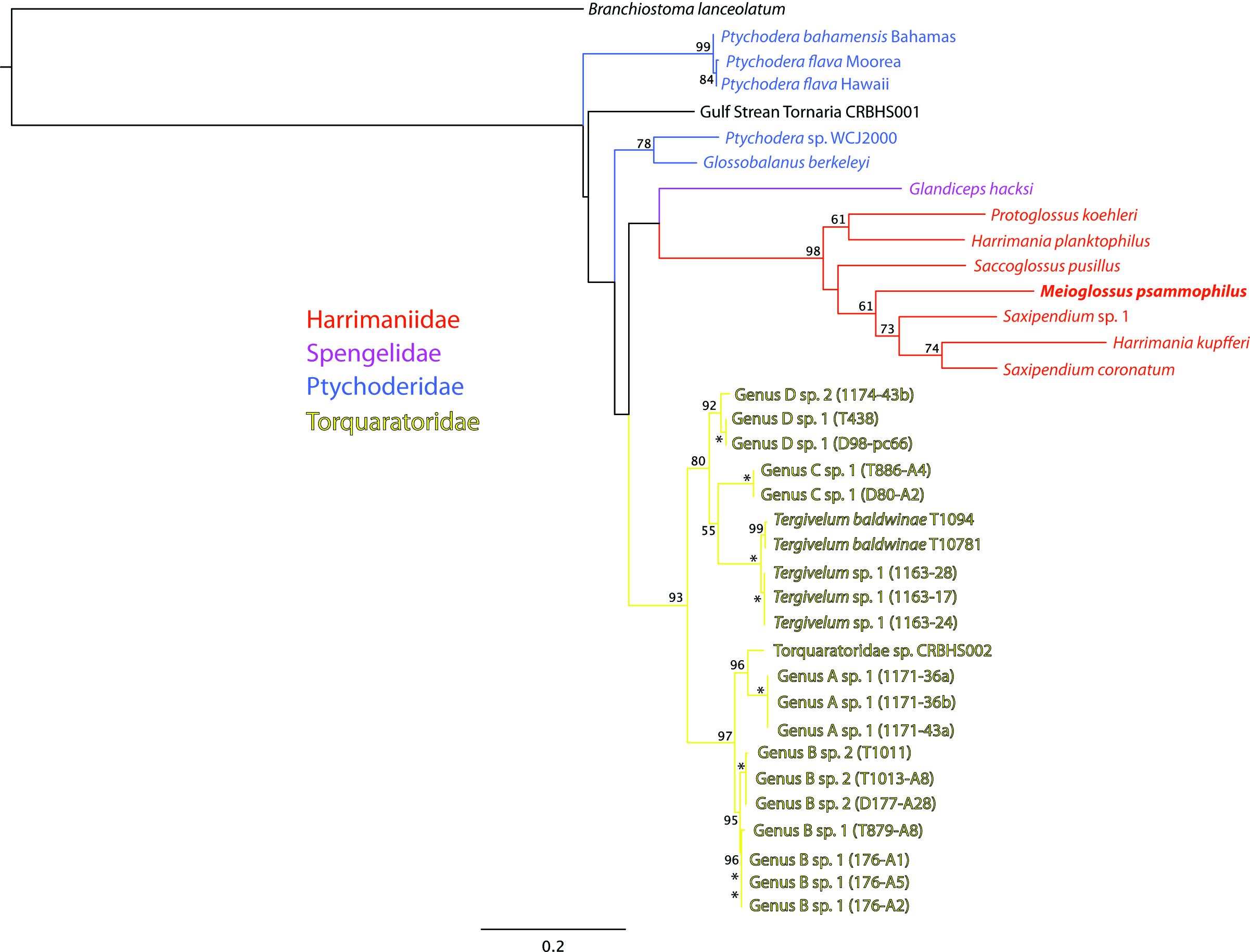

Supplement: Figure S1 — Optimal maximum likelihood tree obtained in RaxML under GTR+Γ for the 16S rRNA data set (− log L = −4667.030968). Values on nodes indicate bootstrap support values above 50%; an asterisk indicates a value of 100%. (TIF) [file pone.0048529.s001.tif]

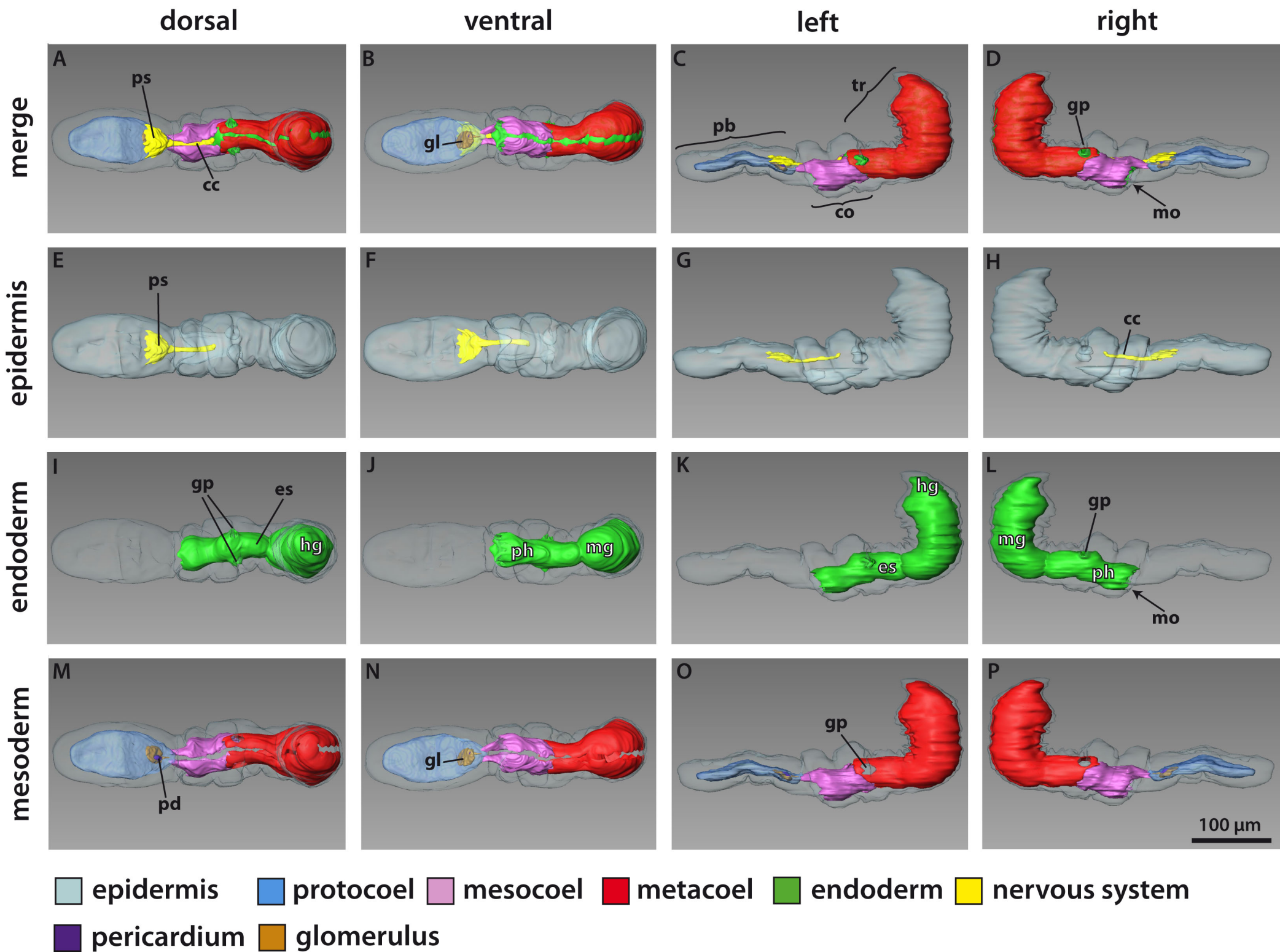

scale bar applicable to all images

Supplement: Figure S2 — Meioglossus psammophilus gen. et sp. nov., 3D-reconstruction of the anatomy of the main organ systems. Click on image to activate 3D model. (PDF) [file pone.0048529.s002.pdf]
